# Prospective assessment of inter-rater reliability of a neonatal adverse event severity scale

**DOI:** 10.3389/fphar.2023.1237982

**Published:** 2023-09-07

**Authors:** Thomas Salaets, Thierry Lacaze-Masmonteil, Isamu Hokuto, Cheri Gauldin, Amjad Taha, Anne Smits, Liesbeth Thewissen, Ilse Van Horebeek, Armuchou Shoraisham, Khorshid Mohammad, Manami Suzuki, Shiori Komachi, Kurt Michels, Mark A. Turner, Karel Allegaert, Tamorah Lewis

**Affiliations:** ^1^ Department of Development and Regeneration, KU Leuven, Leuven, Belgium; ^2^ Department of Pediatrics, Cumming School of Medicine, University of Calgary, Calgary, AB, Canada; ^3^ Department of Pediatrics, St. Marianna University School of Medicine, Kawasaki, Japan; ^4^ Department of Pediatrics, Kansas Children’s Mercy Hospital, University of Missouri Kansas City School of Medicine, Kansas City, MO, United States; ^5^ Neonatal Intensive Care Unit, University Hospitals Leuven, Leuven, Belgium; ^6^ Neonatal Intensive Care Unit, St. Augustinus, GZA Hospitals, Antwerp, Belgium; ^7^ Critical PATH Institute, Tucson, AZ, United States; ^8^ Institute of Lifecourse and Medical Sciences, Liverpool Health Partners, University of Liverpool, Liverpool, United Kingdom; ^9^ Neonatal Unit, Liverpool Women’s Hospital, Liverpool, United Kingdom; ^10^ Department of Pharmaceutical and Pharmacological Sciences, KU Leuven, Leuven, Belgium; ^11^ Department of Hospital Pharmacy, Erasmus MC, Rotterdam, Netherlands; ^12^ Division of Clinical Pharmacology and Toxicology, Hospital for Sick Children, Toronto, ON, Canada

**Keywords:** adverse event (AE), clinical trial, data standards, drug development, drug safety, neonatal

## Abstract

**Introduction:** To ensure the quality of clinical trial safety data, universal data standards are required. In 2019 the International Neonatal Consortium (INC) published a neonatal adverse event severity scale (NAESS) to standardize the reporting of adverse event (AE) severity. In this study the reliability of AE severity grading with INC NAESS was prospectively assessed in a real-world setting.

**Methods:** Severity of AEs was assessed by two independent observers at each of four centers across the world. In each center two series of 30 neonatal adverse events were assessed by both observers: in a first phase with a generic (Common Terminology Criteria for Adverse Events, CTCAE) severity scale not specific to neonates, and in a second phase with INC NAESS (after a structured training). Intraclass correlation coefficients (ICC) were calculated to express inter-rater agreement in both phases, and bootstrap sampling was used to compare them.

**Results:** 120 AEs were included in each of both phases. The ICC with the use of INC NAESS in phase 2 was 0.69. This represents a significant but modest improvement in comparison to the initial ICC of 0.66 in phase 1 (confidence interval of ratio of ICC in phase 2 to phase 1 = 1.005–1.146; excludes 1). The ICC was higher for those AEs for which a diagnosis specific AE severity table was available in INC NAESS (ICC 0.80).

**Discussion:** Good inter-rater reliability of the INC NAESS was demonstrated in four neonatal intensive care units (NICUs) across the globe. The ICC is comparable to what is reported for scales with similar purposes in different populations. There is a modest, but significant, improvement in inter-rater agreement in comparison to the naïve phase without INC NAESS. The better performance when reviewers use AE-specific NAESS tables highlights the need to expand the number of AEs that are covered by specific criteria in the current version of INC NAESS.

## Introduction

Accurate reporting of adverse events (AEs) is a prerequisite for a solid safety analysis in any clinical trial and the responsibility of investigators, sponsors and clinicians (Davis et al., 2020). Besides an evaluation of seriousness, expectedness and causality, a severity grade can be assigned to an AE, offering a more layered appreciation of its medical intensity or impact (International and Brouder, 2009). In order to make this information interpretable for sponsors and regulatory authorities as well as comparable between centers, countries and trials, standardized AE severity scales have been developed as a common language (Kush and Goldman, 2014). Until recently, such a scale was not available for the neonatal population.

In 2019, the International Neonatal Consortium (INC), a multistakeholder organization engaged in neonatal research (academia, industry, regulatory authorities, nursing and parent representatives) developed and published a neonatal adverse event severity scale (NAESS) to standardize the reporting of severity in this high-risk population (Salaets et al., 2019). The scale contains a generic neonatal AE severity grading table that uses criteria relevant to neonates to define severity of any possible AE. It also contains diagnosis-specific severity grading criteria for a set of 35 typical and common neonatal AEs. The instrument is publicly available under “INC Terminology” through the Thesaurus of the US National Cancer Institute (National Institutes of Health, 2023) and has been linked to terms from the Medical Dictionary for Regulatory Activities (MedDRA) (Brown et al., 1999). This recent initiative parallels the longer existing severity scales in other patient populations and research fields (Common Terminology Criteria for Adverse Events CTCAE, 2023; FDA, 2023; National Institute of Allergy and Infectious Diseases, 2023), but uses criteria that are readily applicable to neonates and their common AEs. The NAESS scale is unique in that it accounts for baseline clinical status of (critically ill) infants in the hospital setting, particularly in the neonatal intensive care unit (NICU)s, where many clinical trials are conducted.

An AE severity scale is typically a consensus document. It aims to reduce interobserver variability in AE severity assessments, however for few of the existing instruments there is empirical data available to support this hypothesis (Atkinson et al., 2012; Rampersaud et al., 2016). For INC NAESS a retrospective validation study based on historical case report forms has recently been published (Lewis et al., 2021). This study demonstrated moderate to good reliability of the scale (intraclass correlation coefficient, ICC = 0.63). The results highlighted a need for training of AE assessors and more complete prospectively collected data. The current study represents a complementary prospective effort, that takes the shortcomings of the retrospective validation into account by including (Davis et al., 2020) structured training of NAESS and (International and Brouder, 2009) severity assessment of AEs in a prospective real-time neonatal intensive care unit (NICU) setting. We hypothesized that the use of INC NAESS would improve the interrater reliability of AE severity assessment, in comparison to the current standard without a neonate-specific severity scale.

## Materials and methods

This is an international multicenter study involving four neonatal intensive care units (University Hospitals in Leuven, Belgium; St. Marianna Medical University Hospital in Kawasaki, Japan; Alberta Children’s Hospital in Calgary, Canada and Children’s Mercy in Kansas City, United States). The study was approved by each center’s ethical review board and was conducted in concordance with the Declaration of Helsinki.

In each center a two-phased prospective observational study was conducted between February 2020 and November 2021. For each of the two phases, 30 AEs that occurred in neonates below 44 weeks of postmenstrual age (both in routine clinical care or in clinical trials) during admission in the NICU were identified at each of the participating centers. An AE was defined as “any untoward medical occurrence in a patient or clinical trial participant administered a medicinal product and which does not necessarily have a causal relationship with this product” (International and Brouder, 2009). Case selection was pragmatic and based on the availability of the study team and the recognition of events by the nurses and clinical team on the NICU. A dedicated person (“case identifier”) ensured a variety of severity and pathology. The goal was to include 20 events for which diagnosis-specific severity criteria were available in INC NAESS version 1, and 10 other events (for which only generic criteria were available). Only one AE was included per patient.

In each center, two observers that remained fixed throughout the study graded the severity of the AEs independently of each other. Within 72 h after identification of the case, they were asked to grade the severity of the AE on a 5-point scale (mild, moderate, severe, life-threatening or death) and data were recorded in a REDCap database (Vanderbilt University, Nashville, TN). Both observers were able to assess severity at the bedside and had access to all available information in the electronic health records and the observations of the bedside clinical staff.

In a first phase (30 cases) the observers were not given any specific guidance on how to assign severity grades other than the generic non-neonatal severity table of Common Terminology Criteria for Adverse Events (CTCAE, used in oncology clinical trials) (Common Terminology Criteria for Adverse Events CTCAE, 2023). The absence of specific neonatal guidance reflects current real-world practices. After completion of phase 1, all observers received a copy of the INC NAESS, together with a training module. The 30-min training module (.ppt-format) consisted of general information on how to apply INC NAESS and several examples of adverse event severity gradings. The training module can be found in Supplementary Material. In phase 2 (30 cases), the trained observers had the INC NAESS available for severity grading. For the Japanese site, a Japanese translation had been developed with a reverse-translation to English by an independent interpreter to ensure the translation quality. All other 3 sites worked with the original English version. The generic severity criteria and an example of specific severity criteria (e.g., infantile apnea) are visualized in Tables 1, 2. For the full list of specific criteria and for the Japanese translation we refer to the NCI Thesaurus (National Institutes of Health, 2023).

**TABLE 1:** *The generic severity criteria in INC NAESS* (Salaets et al., 2019).

Grade 1	Grade 2	Grade 3	Grade 4	Grade 5
Mild	Moderate	Severe	Life-threatening	Death
Generic severity criteria				
Mild; asymptomatic or mild symptoms; clinical or diagnostic observations only; no change in baseline age-appropriate behavior*; no change in baseline care or monitoring indicated	Moderate; resulting in minor changes of baseline age-appropriate behavior*; requiring minor changes in baseline care or monitoring***	Severe; resulting in major changes of baseline age-appropriate behavior* or non-life threatening changes in basal physiological processes**; requiring major change in baseline care or monitoring****	Life-threatening; Resulting in life-threatening changes in basal physiological processes**; requiring urgent major change in baseline care	Death related to AE

*Age-appropriate behavior refers to oral feeding behavior, voluntary movements and activity, crying pattern, social interactions and perception of pain.

**Basal physiological processes refer to oxygenation, ventilation, tissue perfusion, metabolic stability and organ functioning.

***Minor care changes constitute: brief, local, non-invasive or symptomatic treatments.

****Major care changes constitute: surgery, addition of long term treatment, upscaling care level If the different factors of this scale result in conflicting severity grades, the highest grade should be reported.

**TABLE 2:** *An example of specific severity criteria (e.g., apnea of prematurity). For the full list of specific criteria we refer to the NCI Thesaurus* (National Institutes of Health, 2023) *or the initial publication on INC NAESS* (Salaets et al., 2019).

Infantile apnea				
Definition C154938 │10077322: Cessation of air flow				
Self-limiting apnea	Apnea responsive to stimulation or intermittent FiO2-increase	Apnea requiring stimulation and sustained FiO2 increase; requiring non-invasive ventilation; reoccurrences requiring start of or relevant increase in dose of respiratory stimulants or other major care changes	Life-threatening respiratory and/or hemodynamic compromise; (semi-)urgent intubation required	Death

Results are described as levels of agreement between the two observers. Absolute agreement means that both observers documented the same severity grade, and their levels are expressed as a proportion of the total number of cases. To summarize interobserver variability, intraclass correlation coefficients (ICC) were calculated using a two-way random model for absolute agreement with single measures (ICC_2,1_) for both phases across all centers and AE types. Our primary hypothesis was that the ICC would increase between phase 1 and phase 2. To test that hypothesis, we performed bootstrap sampling (10.000 samples) to calculate the confidence interval for the ratio of the two ICCs and test whether the ratio differs from 1 (that is, whether the ICC changes from phase 1 to phase 2, with the introduction of INC NAESS). In an exploratory analysis ICC’s per center and per AE type (generic table versus AE-specific table) were calculated and compared using the same methodology. For all statistical analyses the IRR package in R (Vienna University of Economics and Business, Vienna, Austria) was used.

In an *a priori* power analysis it was calculated that, with a pooled number of 120 cases in each phase (i.e., 4 centers with 30 cases in each phase) and an estimated ICC of 0.5–0.7 [comparable to published reliability data on AE severity scales (Atkinson et al., 2012; Rampersaud et al., 2016)], a rather narrow confidence interval of <0.25 would be obtained.

## Results

Over the four centers, a total of 240 AEs were assessed, each by two observers. Of these 240, 171 were events for which AE-specific severity criteria were available and 69 involved diagnoses for which the generic severity criteria had to be used. The full list of AEs included in each phase can be found in Supplementary Table S1. In one center (US) the two observers were research nurses, in the other three centers (Canada, Japan, Belgium) they were both staff neonatologists.

**FIGURE 1:**
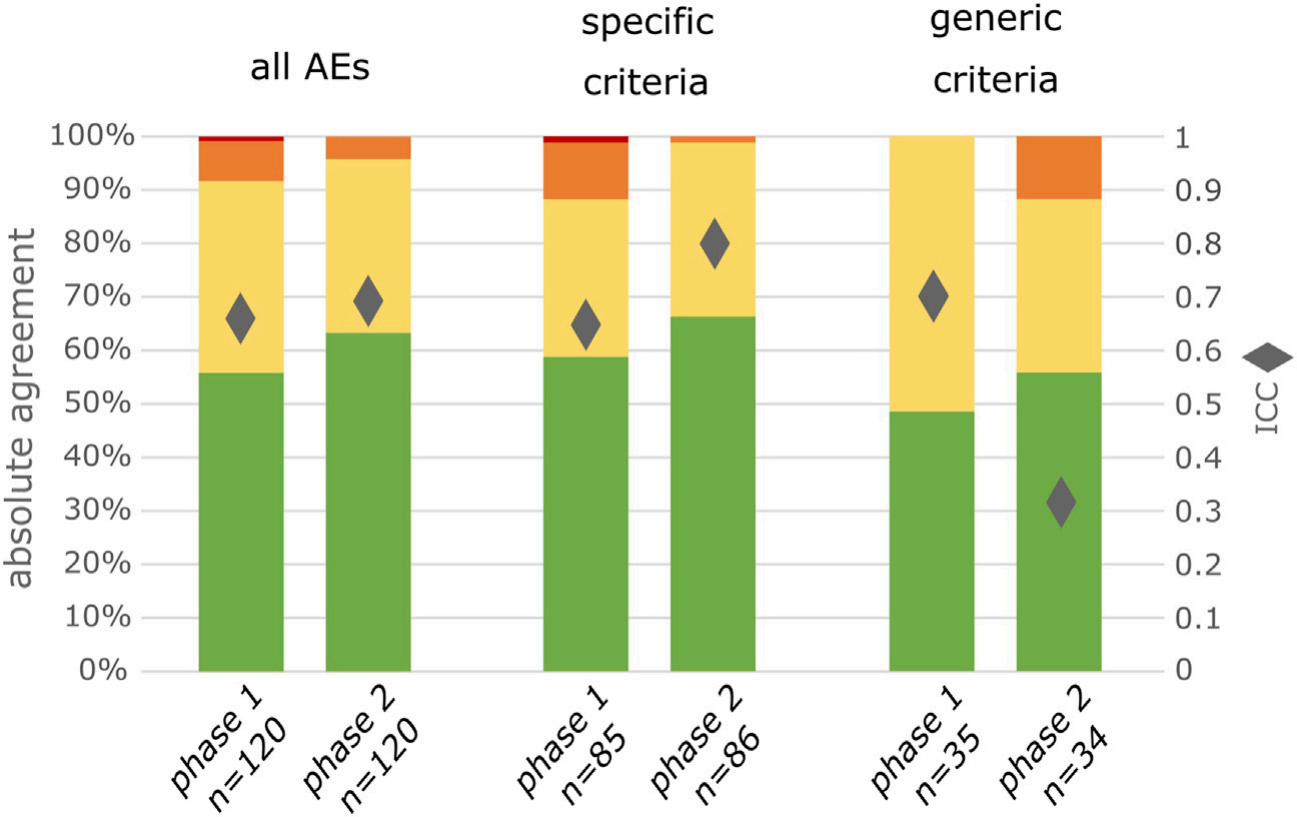
Degree of agreement between 2 observers for all included AEs, for exclusively the AEs for which specific criteria are available in INC NAESS and for exclusively the AEs for which the generic criteria had to be used. Green represents complete agreement, yellow a 1 severity grade difference between observers, orange a 2 severity grades difference and red a 3 severity grades difference. The diamonds represent the ICC values.

In phase 1, there was absolute agreement between observers on the severity grade in 67/120 (56%) of AEs. In phase 2, with the use of INC NAESS, there was absolute agreement in 76/120 (63%) of AEs (Figure 1). This corresponds respectively to an ICC of 0.66 in phase 1 and 0.69 in phase 2. Using the predefined bootstrap sampling method this improvement in interobserver agreement is statistically significant (CI of ratio of ICC in phase 2 versus phase 1 = 1.005-1.146; excludes 1).

For those AEs for which specific criteria were available in INC NAESS, absolute agreement and ICC increased from phase 1 to phase 2 (50/85 to 57/86 or 59%–66% for absolute agreement; 0.65 to 0.80 for ICC; Figure 1). This is a statistically significant increase (CI of ratio of ICC in phase 2 versus phase 1 = 1.193–1.341). For the other AEs, for which INC NAESS does not provide specific criteria and the generic table had to be used, the agreement was lower than that of the AEs graded with diagnosis-specific tables. It was lower in phase 1 and increased in phase 2 to a level only below that of the AEs with specific criteria (17/35 to 19/34 or 48%–55%; Figure 1). The ICC for these AEs graded with the generic table however decreased from 0.70 to 0.32, which is also significant (CI of ratio of ICC in phase 2 versus phase 1 = 0.420–0.544). This is likely due to an increased number of cases for which the difference between the severity grades of both observers was 2 (Figure 1).

**FIGURE 2:**
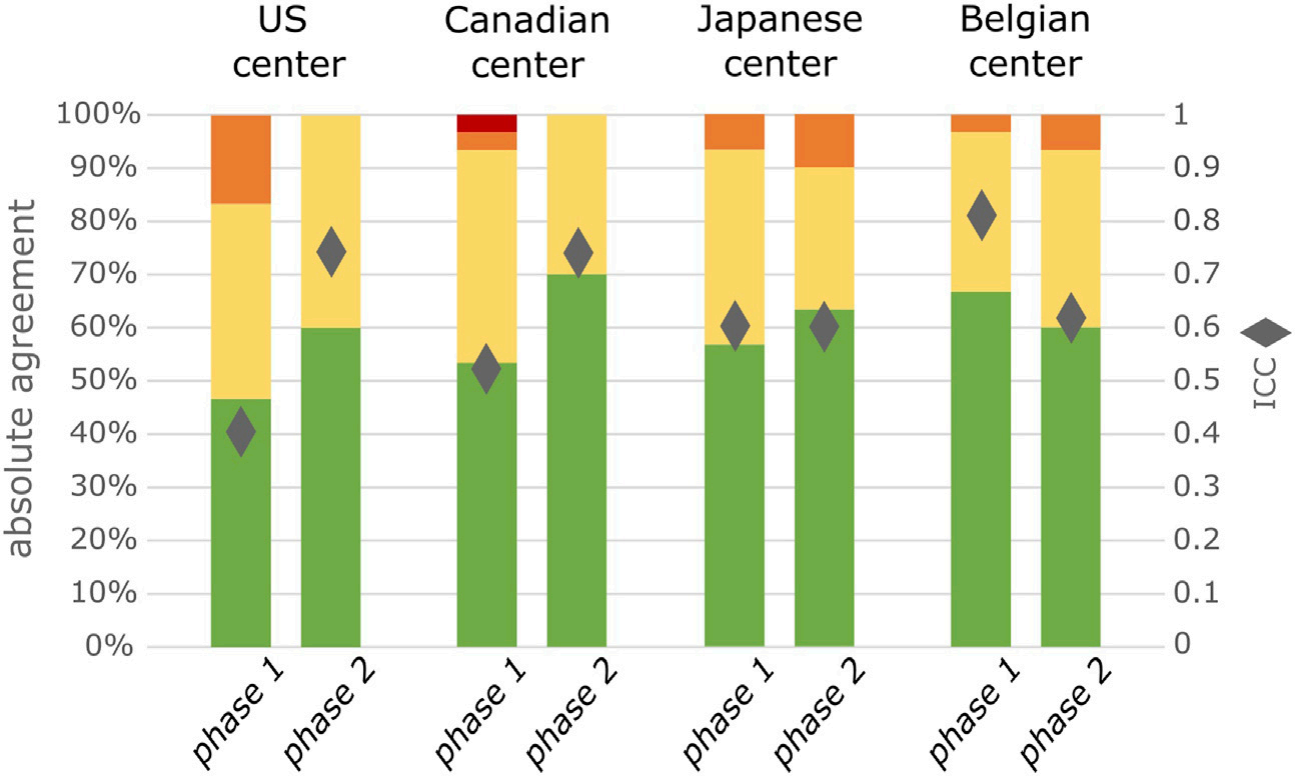
Degree of agreement between 2 observers for all included AEs separated by center, (*n* = 30 per phase per center). Green represents complete agreement, yellow a 1 severity grade difference between observers, orange a 2 severity grades difference and red a 3 severity grades difference. The diamonds represent the ICC value.

In an additional analysis, we observed that the ICC increased significantly between phases in both center 1 (United States; CI 1.714–2.180) and center 2 (Canada; CI 1.343–1.647). It was not significantly different in the center 3 (Japan; CI 0.956–1.087) and decreased in center 4 (Belgium; CI 0.737–0.822). An increase in absolute agreement between phase 1 and phase 2 can however be observed in 3/4 centers (Figure 2). When comparing those AEs graded with specific severity criteria in phase 2 to the same type of AEs in phase 1, there was an increase in absolute agreement in 3/4 centers while it was equal in 1 center. For the AEs graded with generic criteria absolute agreement increased in 2/4 centers while it was equal in 1 and decreased in the other 1 (Supplementary Table S2.)

## Discussion

In this study, we prospectively assessed the reliability of AE severity grading with the INC NAESS in a real-world setting. The ICC of the scale in this setting was estimated at 0.69, which is considered good inter-rater agreement (Cicchetti, 1994). When specifically examining the performance of the scale for only those AEs for which specific severity criteria were available, the ICC further increased to 0.80, reflecting excellent inter-rater agreement (Cicchetti, 1994).

This estimate is in line with the limited published data on inter-rater agreement of severity grading with AE severity scales used in other populations. Atkinson et al. (2012) measured inter-rater agreement for some specific AEs from the CTCAE severity scale for oncology trials. Two clinicians assessed severity independently in oncology outpatients in a real-life setting, resulting in ICCs for these specific AE severity scales ranging from 0.46 to 0.71. Another very specific instrument for evaluation of AEs after spinal surgery was in a prospective real-life setting reported to have an ICC of 0.75 (Rampersaud et al., 2016). For most AE severity scales that are commonly used and recommended (e.g., most AE criteria in CTCAE, DAIDS (Division of Acquired Immunodeficiency Syndrome), FDA (USA Food and Drug Administration) toxicity table for healthy volunteers in vaccine trials) there are however no empirical data available evaluating reliability.

Our study was complementary to a previously published retrospective reliability study on INC NAESS. In that study, 60 AE case report forms from a recent neonatal clinical trial were assigned to 12 observers for independent severity grading without having access to the full electronic health record or without being able to directly examine the patient. Under these circumstances, which mimics the severity grading process as currently done at the level of a safety monitoring board, sponsor or regulator, the ICC of INC NAESS was estimated at 0.63 (Lewis et al., 2021). This is slightly lower than what was measured in this prospective study.

A possible factor for the higher ICC in the prospective study is that case report forms might not always contain all information that is needed for severity assessment with the INC NAESS criteria, while in the prospective study both observers had access to all information in real-time and at bedside. On the other hand, a setting in which observers have access to a limited, but equal, summary of the event available might also overestimate inter-rater agreement in comparison to a real-life setting where severity assessment is influenced by an observer’s personal clinical impression, exact timing and thoroughness of the case review, etc... We hypothesize that the most ideal reliability would be obtained in a setting where structured case report forms summarize and standardize the data to which the assessors of severity are exposed, whilst ensuring that all elements necessary for severity assessment are available. A severity assessment can only be as good as the quality of the observations and information gathered by bedside care providers on which it is based. Further progress in standardization of safety information in neonates could likely be made by developing new digital tools that aid extraction of clinical data from the electronic health record and structure reporting of AE severity, without increasing the administrative burden. This could include the definition of core data concepts of AE severity (in parallel to more broad core neonatal data concepts (Molloy et al., 2018; Webbe et al., 2020)), the development of layered electronic case report forms (eCRFs) to facilitate and guide the collection of key information and maybe even (AI-driven) methods to automatically populate information from not-structured sources such as health records.

In this prospective study, we not only measured the inter-rater agreement between observers that were trained with INC NAESS, we also compared it to a naïve setting that is comparable to how AE severity is currently assessed in most clinical trials. This resulted in a significant, however only modest improvement of the ICC (0.66 in phase 1 versus 0.69 in phase 2). The magnitude of this effect is likely underestimated by an observer bias or Hawthorne effect (Paradis and Sutkin, 2017). This term describes the altered behavior of a study subject that is aware of being observed. This study ran over a relatively long period of time with phase 1 and phase 2 consecutively, and we suspect observers might have been more cautious in their assessment of AE severity especially at the start of the study, overestimating agreement in a naïve real-world setting. This is specifically clear in center 4 where ICC decreased between phase 1 and phase 2, but where the inter-rater agreement in the naïve phase 1 was unusually high in comparison to the other centers (Figure 2).

This study did involve a short training on the use of INC NAESS. This likely contributes to the slightly better ICC than reported in the retrospective study. However we did not measure the adherence to the intended use of INC NAESS as explained in the module and we did only train the observers that assigned the severity grade and not the bedside clinical team that records most of the data in the electronic health record. We also did not test the performance of this specific training module and improving the training modalities could potentially result in a more important increase in inter-rater agreement. The INC NAESS training module that was developed for this study can be found in Supplementary Material. A web-based version of a training tool for future end-user education is currently being developed.

Even if the use of INC NAESS improves the reliability of severity assessment only modestly, it also should improve the validity of the severity estimate. Reliability refers to the difference between two observers while validity refers to how close the estimates are to the absolute truth. A very good inter-rater agreement within one center, can for instance mean that two observers that might have been trained in a similar way and that work together closely in clinical care, have a similar intuition about severity of events, but it does not necessarily mean that it is close to how people with a very different background perceive it. The latter is impossible to measure as there is no absolute gold standard. Furthermore this study was set-up in such a way that it only assesses agreement between observers within a center. Nevertheless we think that the INC NAESS does add an important but unmeasurable factor of validity to AE severity grading. The availability of a shared definition standardizes severity information universally and their specificity to (critically ill) neonates ensures that this information is meaningful in this particular population.

In the exploratory analyses of this study we did observe a difference between centers in how INC NAESS affected the reliability of severity grading. A partial explanation for this might be the difference in observer background. The US center, which had the highest ICC in phase 2, and the largest improvement in ICC between the two phases, was the only center in which the 2 observers were research nurses. In all other centers the 2 observers were neonatologists. Additionally, there might by a language effect with the 2 English speaking sites, using the original English version of INC NAESS, having the highest interobserver agreement in phase 2. Again, these relative differences between centers only reflect differences in inter-rater reliability, not necessarily the validity of the severity estimates.

Finally, we observed a clear difference in reliability between the specific criteria (ICC 0.80) and the generic criteria (ICC 0.32) of INC NAESS. This can be explained as the specific criteria are more applied and contain very specific descriptors of a given AE (i.e., apnea or seizures), and are therefore easier to use. The generic criteria on the other hand are on purpose very broad and require some interpretation. The current and first version of INC NAESS contains specific severity criteria for 35 common neonatal adverse events, which were chosen based on a stakeholder survey (Salaets et al., 2019). In comparison to v5.0 of CTCAE, which is the severity scale used in oncology trials and which has specific criteria covering 837 AEs this is still rather limited (Common Terminology Criteria for Adverse Events CTCAE, 2023). Major blind spots of the current version of INC NAESS are for instance AEs based on abnormal laboratory values such as altered liver or kidney function. This study clearly highlights the need to expand the number of AEs covered by specific criteria in INC NAESS. The INC is committed to continued improvement of this instrument.

Specifically, AEs based on laboratory values are considered an important gap of the current version of INC NAESS. As recently reported, there are however no generally accepted, actionable reference values for commonly used laboratory values in neonates, while published information on lab values in neonates is sparse, not systematic and incomplete (Allegaert et al., 2022). Data driven approaches are needed to define normality and levels of abnormality (severity grades) in the term and preterm neonatal population.

Several limitations, such as the unavailability of a common case report form to structure the clinical data, the fact that we did not assess adherence to the use of INC NAESS as explained in the training module, the possible presence of a Hawthorne effect and the fact that we only assessed inter-rater agreement between observers from the same center, have already been discussed above. Additionally it is important to stress that an ICC does not represent a fixed characteristic of an instrument or scale, but that it depends also on the specific settings in which it is measured.

Finally, it should be emphasized there is a (legal) difference between AE severity and seriousness. We did not measure interobserver agreement on assessment of “seriousness”, which has a strict legal definition and drives reporting to regulatory bodies. As the globally harmonized definition of “seriousness” (International and Brouder, 2009)—in our opinion–not readily applicable to the NICU setting, we would also expect large variability between centers in which AEs are reported and which are not. It is up to the regulatory authorities now to evaluate whether the availability of a standardized and reliable scale for AE severity, would alter their guidance on reporting of AEs in this specific population.

In conclusion, a prospective real-world study demonstrated good inter-rater reliability of the INC NAESS, which is comparable to—or even better than—what is reported for scales with similar purposes in different populations. The improvement in reliability of severity grading in comparison to an era without neonatal severity criteria, is modest but significant. We hypothesize that there is an important, but unmeasurable, additional benefit on validity of severity estimates which would be highly beneficial for Ethics Boards and Regulatory Authorities that struggle to understand the impact of AEs in high risk populations. Finally we noted significant differences between centers in different countries, possibly due to different backgrounds of observers. We also noted a higher reliability of specific severity criteria in comparison to generic criteria. Future work should focus on expanding the number of neonatal AEs covered by specific criteria in INC NAESS, on creating and distributing NAESS education tools, and on the development of standardized (digital) case report forms that capture essential elements for severity assessment without increasing administrative burden.

## Data Availability

The original contributions presented in the study are included in the article/Supplementary Materials, further inquiries can be directed to the corresponding author.
