# Supplementary material for: Prospective assessment of inter-rater reliability of a neonatal adverse event severity scale
Source: Front Pharmacol. 2023 Sep 7;14:1237982. doi: 10.3389/fphar.2023.1237982 (PMC10512550; doi:10.3389/fphar.2023.1237982)
Supplement: Supplementary file 1 [file Table1.DOCX]

***Supplementary material***

**Prospective Assessment of Inter-rater Reliability of a Neonatal Adverse Event Severity Scale**

Thomas Salaets^*^, Thierry Lacaze-Masmonteil, Isamu Hokuto, Cheri Gauldin, Amjad Taha, Anne Smits, Liesbeth Thewissen, Ilse Van Horebeek, Armuchou Shoraisham, Khorshid Mohammad, Manami Suzuki, Shiori Komachi, Kurt Michels, Mark A. Turner, Karel Allegaert & Tamorah Lewis for the International Neonatal Consortium

*** Correspondence:** Thomas Salaets: thomas.1.salaets@uzleuven.be

***Supplementary table 1.*** *List of all included AEs, per phase.*

| **Diagnosis** | **Phase 1** | **Phase 2** |
| --- | --- | --- |
| **All diagnoses with specific criteria** | **85** | **86** |
| Neonatal Convulsion | 1 | 1 |
| Neonatal Epileptic Seizure | 2 | 2 |
| Neonatal Intraventricular Hemorrhage | 3 | 4 |
| Retinopathy of Prematurity | 2 | 4 |
| Hypoxic Ischemic Encephalopathy | 1 | 2 |
| Periventricular Leukomalacia | 1 | 0 |
| Infant Irritability | 1 | 2 |
| Infant Sedation | 1 | 2 |
| Neonatal Hypotension | 6 | 5 |
| Neonatal Hypertension | 3 | 1 |
| Neonatal Sinus Tachycardia | 1 | 4 |
| Neonatal Sinus Bradycardia | 1 | 4 |
| Neonatal Tachyarrhythmia | 2 | 2 |
| Neonatal Bradyarrhythmia | 3 | 1 |
| Neonatal Edema | 1 | 1 |
| Neonatal Coagulation Disorder | 3 | 2 |
| Neonatal Culture Positive Sepsis | 3 | 4 |
| Neonatal Culture Negative Sepsis | 3 | 5 |
| Infantile Apnea | 1 | 3 |
| Neonatal Respiratory Insufficiency | 3 | 3 |
| Neonatal Respiratory Distress Syndrome | 3 | 1 |
| Neonatal Pulmonary Hemorrhage | 3 | 3 |
| Persistent Pulmonary Hypertension of the Newborn | 2 | 2 |
| Neonatal Pneumothorax | 4 | 2 |
| Bronchopulmonary Dysplasia | 0 | 0 |
| Necrotizing Enterocolitis | 3 | 4 |
| Neonatal Diarrhea | 1 | 3 |
| Infantile Vomiting | 2 | 3 |
| Feeding Intolerance | 3 | 0 |
| Neonatal Gastrointestinal Bleeding | 1 | 1 |
| Neonatal Spontaneous Intestinal Perforation | 4 | 4 |
| Neonatal Constipation | 4 | 3 |
| Neonatal Rash | 6 | 2 |
| Neonatal Administration Site Complication | 3 | 3 |
| Neonatal Fever | 4 | 3 |
| **Other diagnoses** | **35** | **34** |
| Hydrocephaly post IVH | 1 |  |
| Neonatal Opioid Withdrawal | 1 | 1 |
| Sinus thrombosis |  | 1 |
| Cerebellar hemorrhage |  | 1 |
| Persistent ductus arteriosus | 2 | 2 |
| Long QT | 1 |  |
| Anemia | 2 | 2 |
| Polycythemia |  | 2 |
| Thrombocytopenia |  | 1 |
| Transfusion reaction |  | 1 |
| Hyperbilirubinemia | 2 | 2 |
| Cholestasis | 2 |  |
| Elevated liver function tests | 1 |  |
| Hypertriglyceridemia |  | 1 |
| Volvulus | 1 |  |
| Meconium subobstruction | 1 |  |
| Duodenal atresia |  | 1 |
| Inguinal hernia |  | 1 |
| Abdominal distension | 1 |  |
| Gallstone |  | 1 |
| Hypoglycemia | 2 | 1 |
| Hyperglycemia | 2 | 3 |
| Infiltrates on Chest XR | 1 |  |
| Respiratory tract colonization |  | 1 |
| Desaturation |  | 1 |
| Pulmonary interstitial emphysema | 1 |  |
| Pleural effusion | 1 |  |
| Electrolyte disturbance |  | 1 |
| Metabolic acidosis | 1 | 1 |
| Hyponatremia | 3 | 1 |
| Hypernatremia |  | 3 |
| Hypokalemia |  | 1 |
| Hyperkalemia | 1 |  |
| Hypocalcemia | 1 | 1 |
| Polyuria | 1 |  |
| Oliguria | 1 |  |
| Dehydration | 1 | 1 |
| Renal insufficiency | 3 |  |
| Metabolic bone disease | 1 |  |
| Leakage of umbilical vein catheter |  | 1 |
| Thermolability |  | 1 |

**Supplementary table 2.** *Degree of agreement between 2 observers for all included AEs separated by center and by type of AE (with specific criteria available in INC NAESS versus without). Center 1, 2, 3 and 4 are respectively US, Canada, Japan and Belgium based.*

|  | Total number of AEs | Complete agreement | Difference of 1 severity grade | Difference of 2 severity grades | Difference of 3 severity grades |
| --- | --- | --- | --- | --- | --- |
| **Center 1 (phase 1)** | 30 | 14 (47%) | 11 (37%) | 5 (17%) | 0 |
| *Specific criteria* | 25 | 12 (48%) | 8 (32%) | 5 (20%) | 0 |
| *Generic criteria* | 5 | 2 (40%) | 3 (60%) | 0 | 0 |
| **Center 1 (phase 2)** | 30 | 18 (60%) | 12 (40%) | 0 | 0 |
| *Specific criteria* | 25 | 16 (64%) | 9 (36%) | 0 | 0 |
| *Generic criteria* | 5 | 2 (40%) | 3 (60%) | 0 | 0 |
| **Center 2 (phase 1)** | 30 | 16 (53%) | 12 (40%) | 1 (3%) | 1 (3%) |
| *Specific criteria* | 20 | 12 (60%) | 6 (30%) | 1 (5%) | 1 (5%) |
| *Generic criteria* | 10 | 4 (40%) | 6 (60%) | 0 | 0 |
| **Center 2 (phase 2)** | 30 | 21 (70%) | 9 (30%) | 0 | 0 |
| *Specific criteria* | 21 | 14 (67%) | 7 (33%) | 0 | 0 |
| *Generic criteria* | 9 | 7 (78%) | 2 (22%) | 0 | 0 |
| **Center 3 (phase 1)** | 30 | 17 (57%) | 11 (37%) | 2 (7%) | 0 |
| *Specific criteria* | 20 | 12 (60%) | 6 (30%) | 2 (10%) | 0 |
| *Generic criteria* | 10 | 5 (50%) | 5 (50%) | 0 | 0 |
| **Center 3 (phase 2)** | 30 | 19 (63%) | 8 (27%) | 3 (10%) | 0 |
| *Specific criteria* | 20 | 13 (65%) | 6 (30%) | 1 (5%) | 0 |
| *Generic criteria* | 10 | 6 (60%) | 2 (20%) | 2 (20%) | 0 |
| **Center 4 (phase 1)** | 30 | 20 (67%) | 9 (30%) | 1 (3%) | 0 |
| *Specific criteria* | 20 | 14 (70%) | 5 (25%) | 1 (5%) | 0 |
| *Generic criteria* | 10 | 6 (60%) | 4 (40%) | 0 | 0 |
| **Center 4 (phase 2)** | 30 | 18 (60%) | 10 (33%) | 2 (7%) | 0 |
| *Specific criteria* | 20 | 14 (70%) | 6 (30%) | 0 | 0 |
| *Generic criteria* | 10 | 4 (40%) | 4 (40%) | 2 (20%) | 0 |

**Online supplement.** The powerpoint-based training module can be found in the online supplement.
