# Supplementary material for: Prospective assessment of inter-rater reliability of a neonatal adverse event severity scale
Source: Front Pharmacol. 2023 Sep 7;14:1237982. doi: 10.3389/fphar.2023.1237982 (PMC10512550; doi:10.3389/fphar.2023.1237982)
Supplement: Supplementary file 2 [file Presentation1.PPTX]

## Slide 1
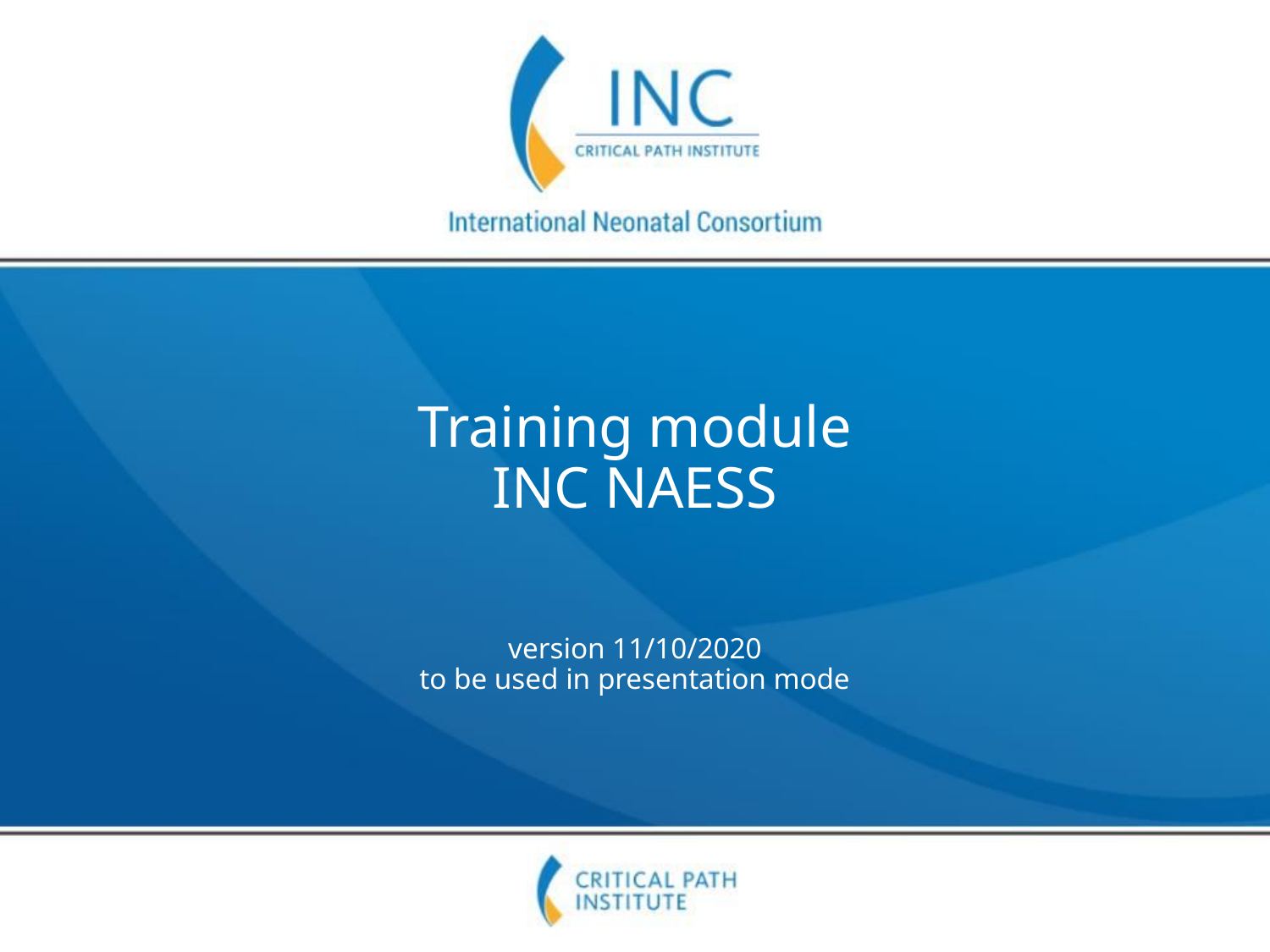

# Training module INC NAESS version 11/10/2020 to be used in presentation mode

## Slide 2
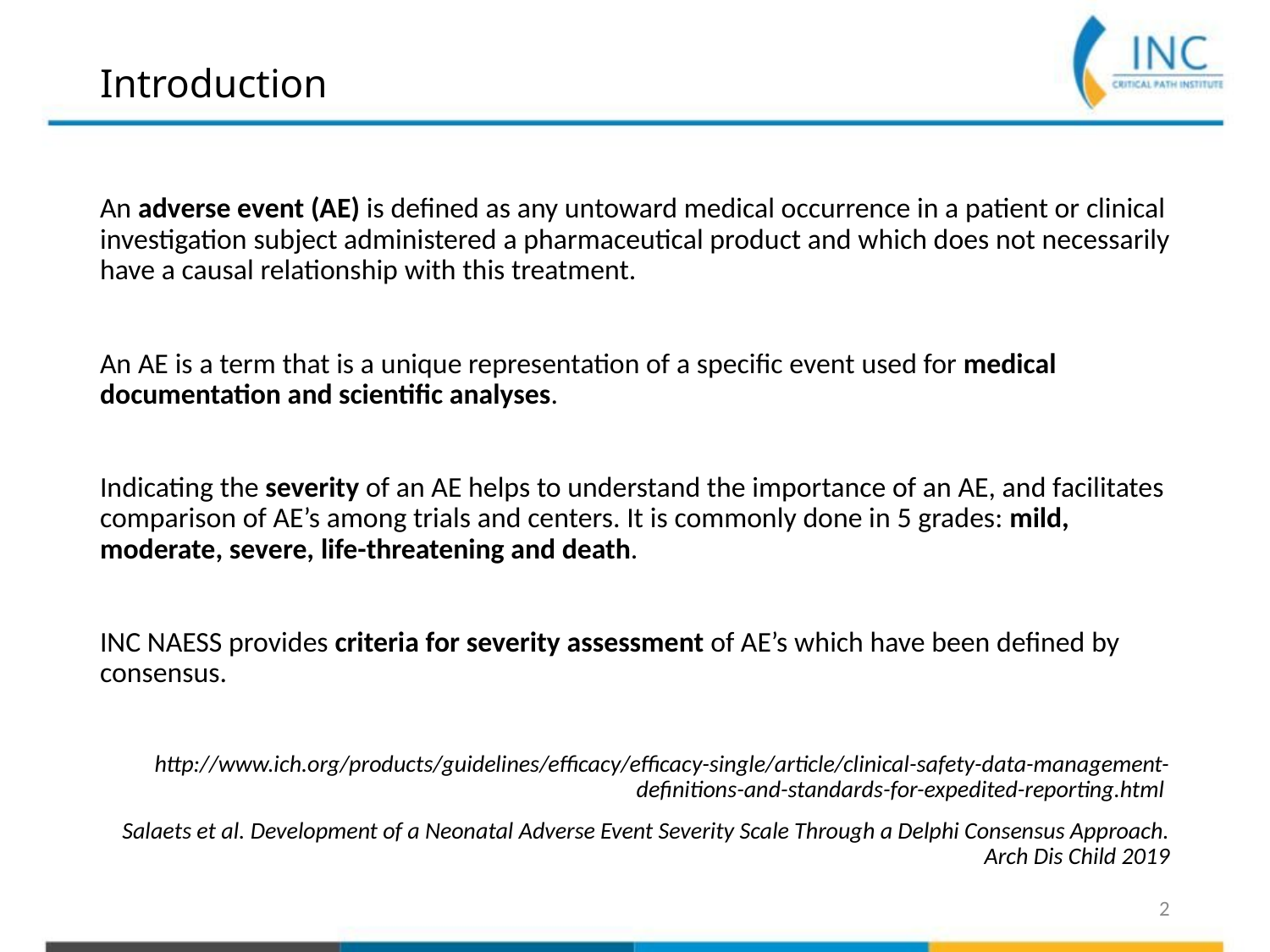

# Introduction
An adverse event (AE) is defined as any untoward medical occurrence in a patient or clinical investigation subject administered a pharmaceutical product and which does not necessarily have a causal relationship with this treatment.
An AE is a term that is a unique representation of a specific event used for medical documentation and scientific analyses.
Indicating the severity of an AE helps to understand the importance of an AE, and facilitates comparison of AE’s among trials and centers. It is commonly done in 5 grades: mild, moderate, severe, life-threatening and death.
INC NAESS provides criteria for severity assessment of AE’s which have been defined by consensus.
http://www.ich.org/products/guidelines/efficacy/efficacy-single/article/clinical-safety-data-management-definitions-and-standards-for-expedited-reporting.html
Salaets et al. Development of a Neonatal Adverse Event Severity Scale Through a Delphi Consensus Approach. Arch Dis Child 2019
2

## Slide 3
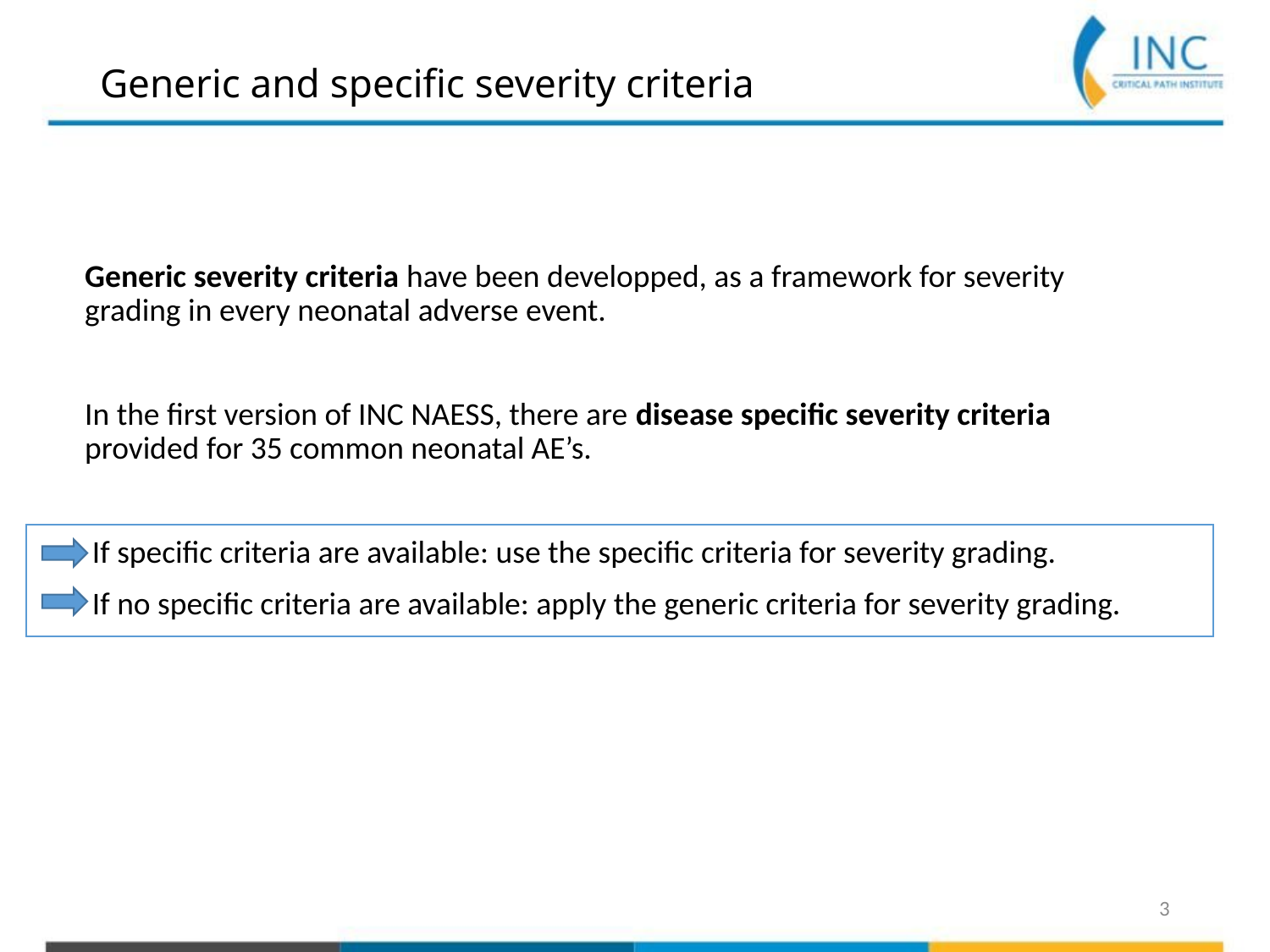

# Generic and specific severity criteria
Generic severity criteria have been developped, as a framework for severity grading in every neonatal adverse event.
In the first version of INC NAESS, there are disease specific severity criteria provided for 35 common neonatal AE’s.
 If specific criteria are available: use the specific criteria for severity grading.
 If no specific criteria are available: apply the generic criteria for severity grading.
3

## Slide 4
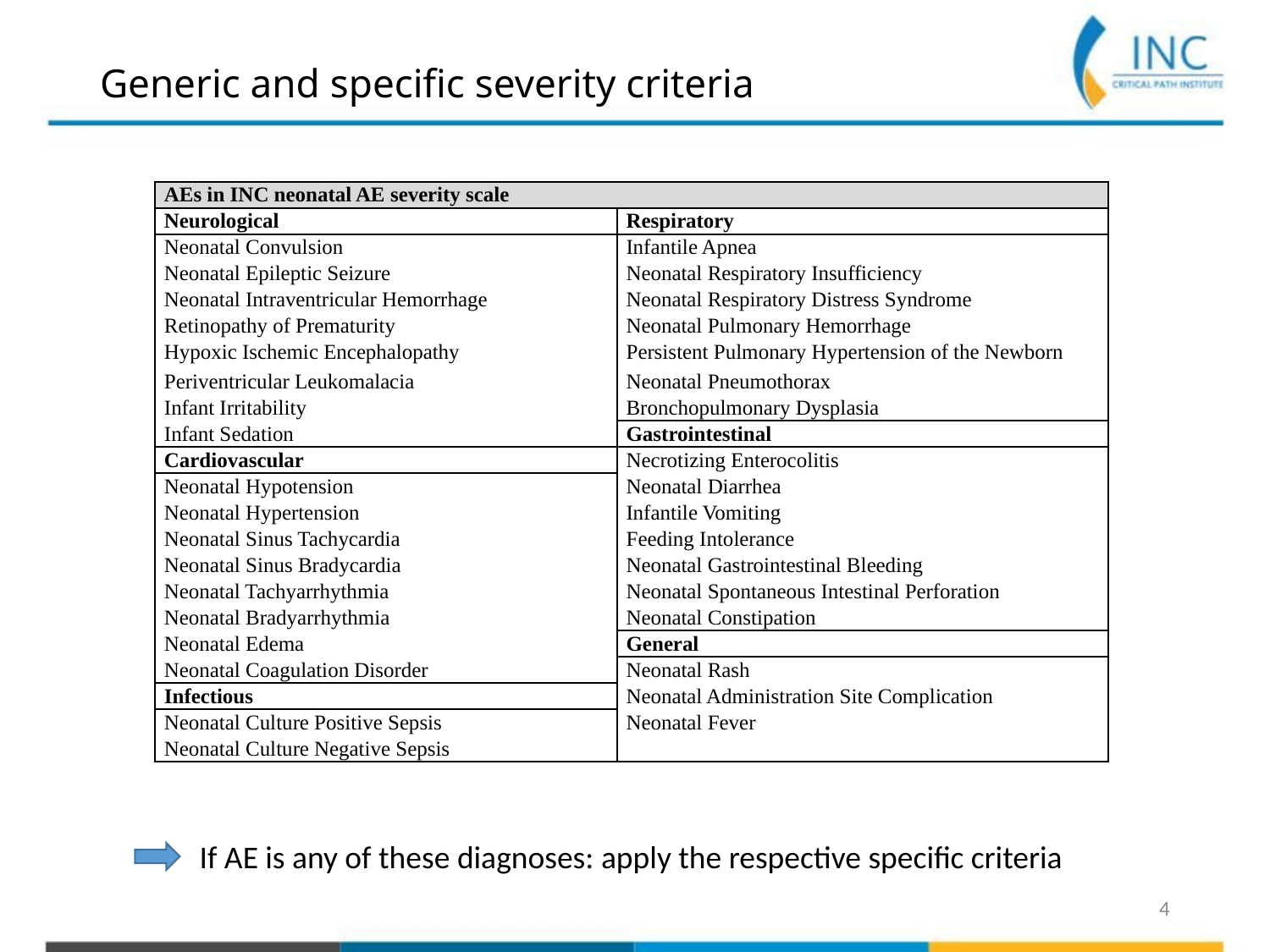

# Generic and specific severity criteria
| AEs in INC neonatal AE severity scale | |
| --- | --- |
| Neurological | Respiratory |
| Neonatal Convulsion | Infantile Apnea |
| Neonatal Epileptic Seizure | Neonatal Respiratory Insufficiency |
| Neonatal Intraventricular Hemorrhage | Neonatal Respiratory Distress Syndrome |
| Retinopathy of Prematurity | Neonatal Pulmonary Hemorrhage |
| Hypoxic Ischemic Encephalopathy | Persistent Pulmonary Hypertension of the Newborn |
| Periventricular Leukomalacia | Neonatal Pneumothorax |
| Infant Irritability | Bronchopulmonary Dysplasia |
| Infant Sedation | Gastrointestinal |
| Cardiovascular | Necrotizing Enterocolitis |
| Neonatal Hypotension | Neonatal Diarrhea |
| Neonatal Hypertension | Infantile Vomiting |
| Neonatal Sinus Tachycardia | Feeding Intolerance |
| Neonatal Sinus Bradycardia | Neonatal Gastrointestinal Bleeding |
| Neonatal Tachyarrhythmia | Neonatal Spontaneous Intestinal Perforation |
| Neonatal Bradyarrhythmia | Neonatal Constipation |
| Neonatal Edema | General |
| Neonatal Coagulation Disorder | Neonatal Rash |
| Infectious | Neonatal Administration Site Complication |
| Neonatal Culture Positive Sepsis | Neonatal Fever |
| Neonatal Culture Negative Sepsis | |
 If AE is any of these diagnoses: apply the respective specific criteria
4

## Slide 5
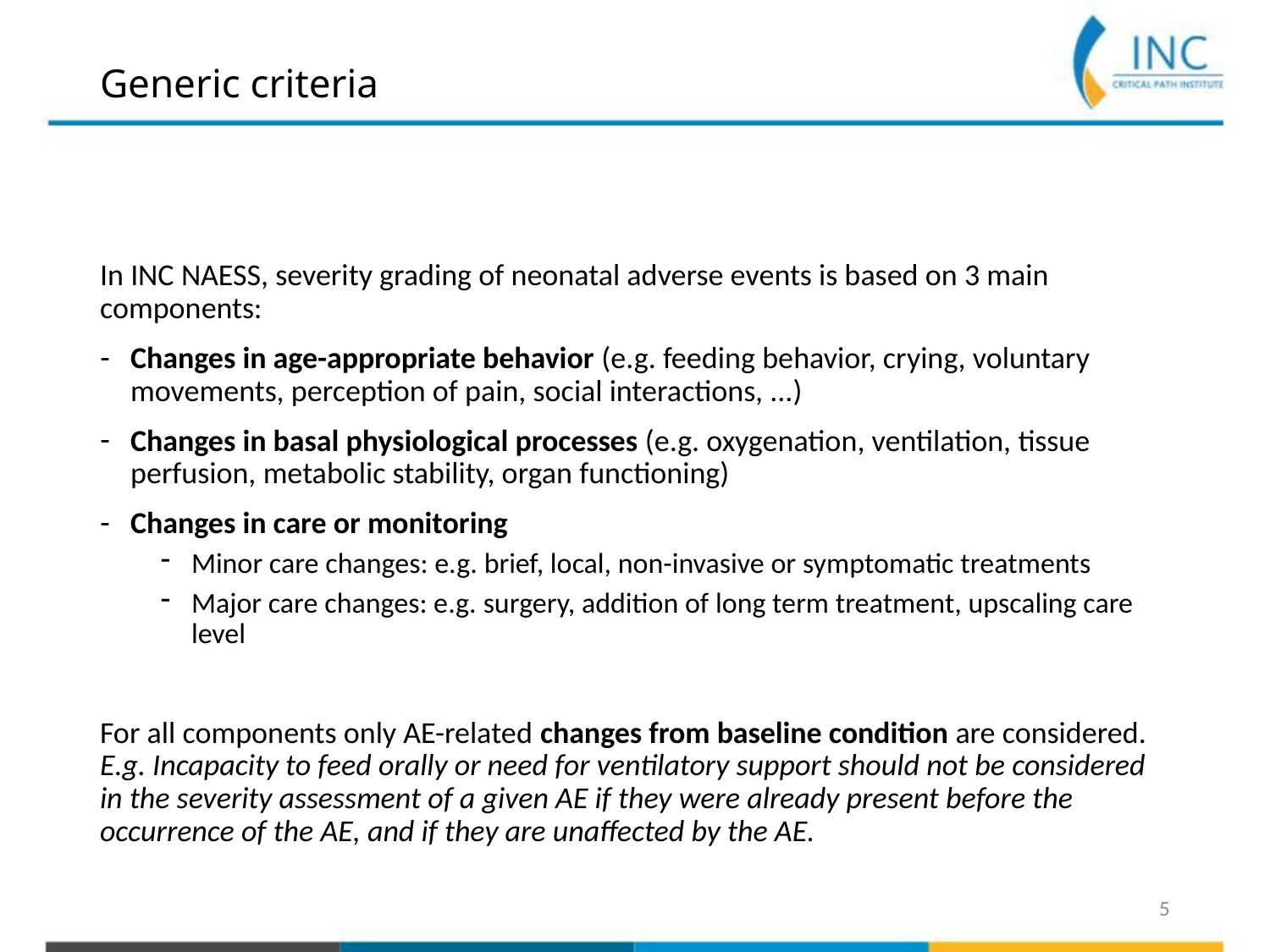

# Generic criteria
In INC NAESS, severity grading of neonatal adverse events is based on 3 main components:
Changes in age-appropriate behavior (e.g. feeding behavior, crying, voluntary movements, perception of pain, social interactions, ...)
Changes in basal physiological processes (e.g. oxygenation, ventilation, tissue perfusion, metabolic stability, organ functioning)
Changes in care or monitoring
Minor care changes: e.g. brief, local, non-invasive or symptomatic treatments
Major care changes: e.g. surgery, addition of long term treatment, upscaling care level
For all components only AE-related changes from baseline condition are considered. E.g. Incapacity to feed orally or need for ventilatory support should not be considered in the severity assessment of a given AE if they were already present before the occurrence of the AE, and if they are unaffected by the AE.
5

## Slide 6
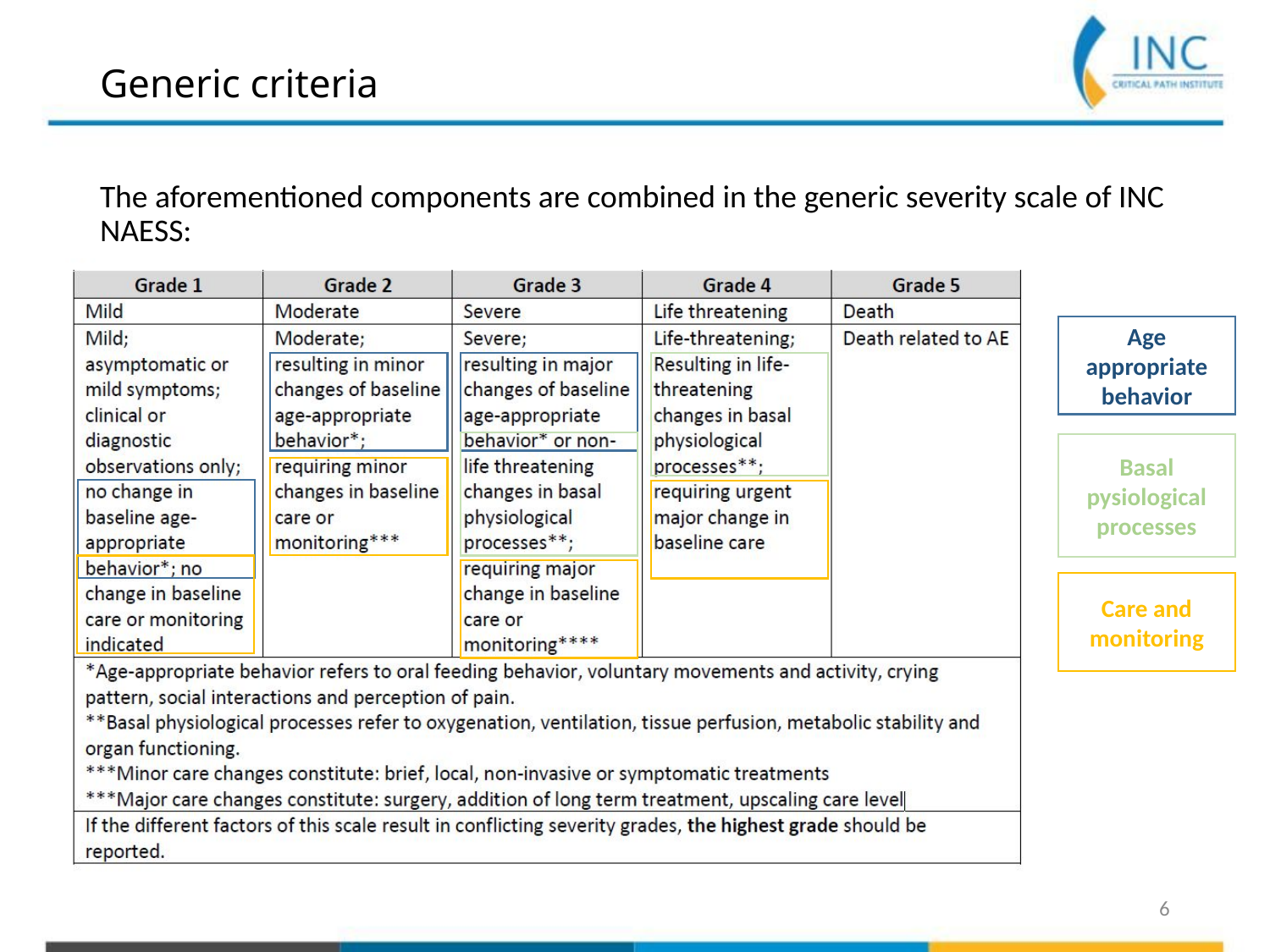

# Generic criteria
The aforementioned components are combined in the generic severity scale of INC NAESS:
Age appropriate behavior
Basal pysiological processes
Care and monitoring
6

## Slide 7
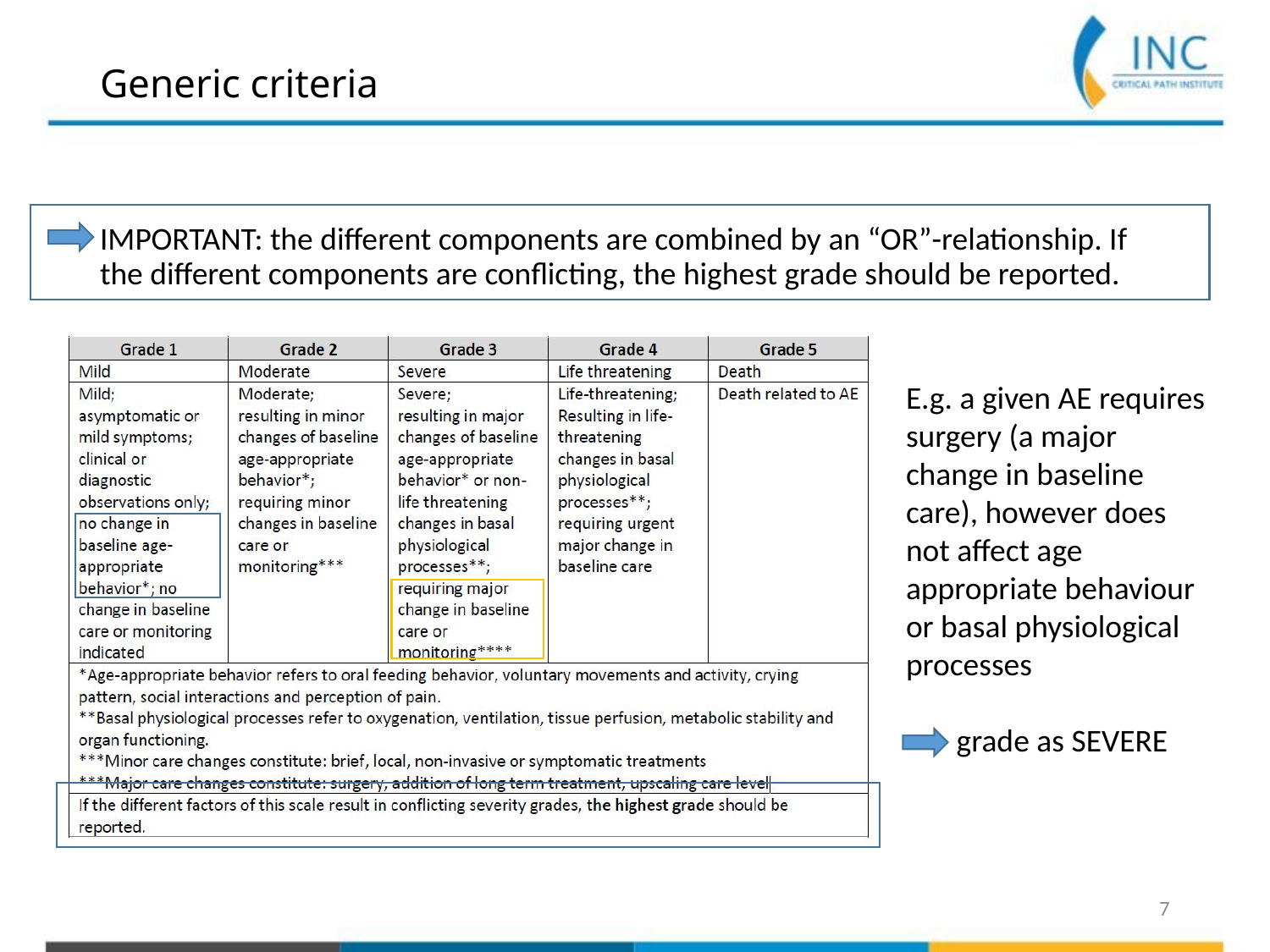

# Generic criteria
IMPORTANT: the different components are combined by an “OR”-relationship. If the different components are conflicting, the highest grade should be reported.
E.g. a given AE requires surgery (a major change in baseline care), however does not affect age appropriate behaviour or basal physiological processes
 grade as SEVERE
7

## Slide 8
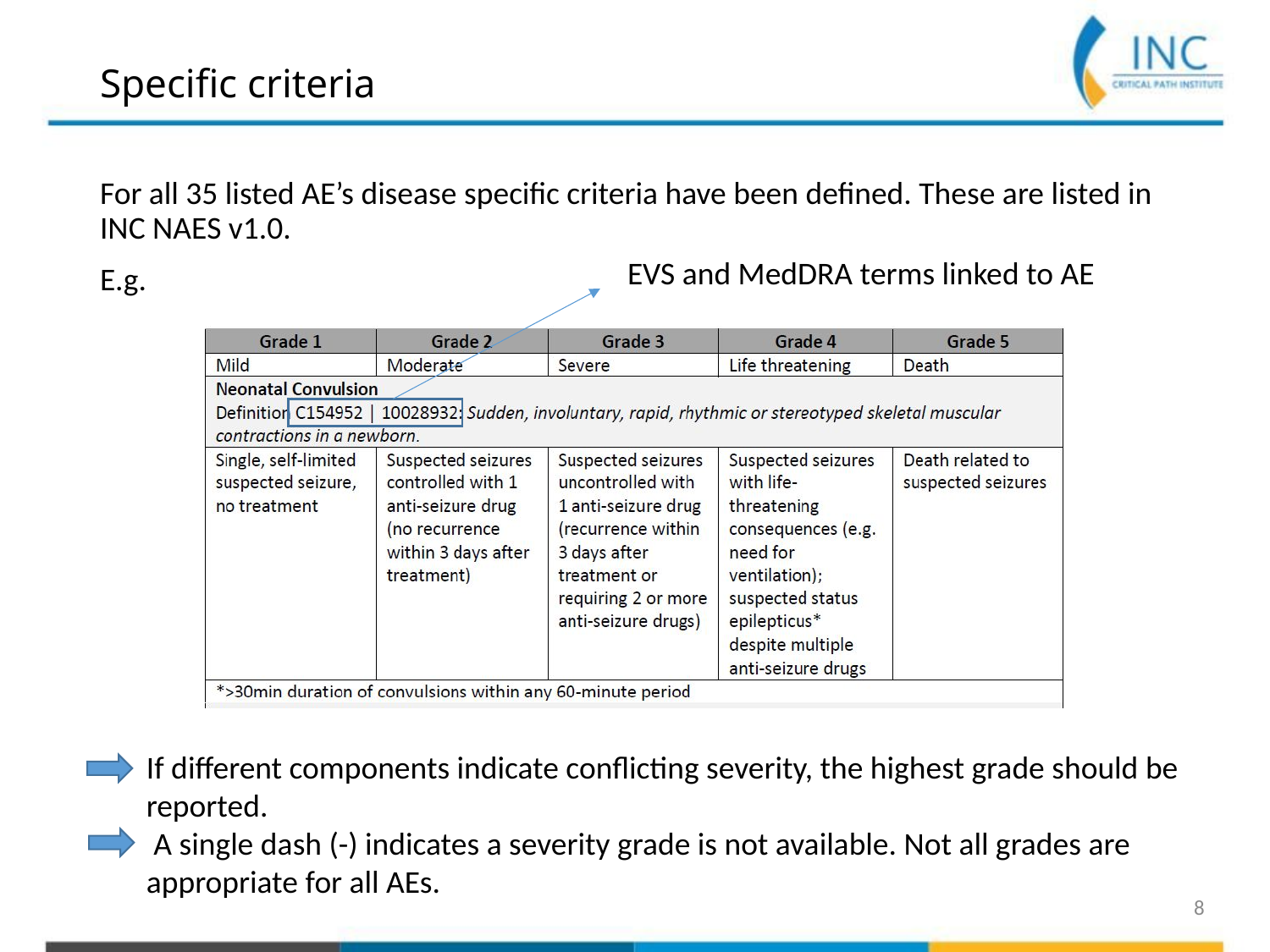

# Specific criteria
For all 35 listed AE’s disease specific criteria have been defined. These are listed in INC NAES v1.0.
E.g.
EVS and MedDRA terms linked to AE
If different components indicate conflicting severity, the highest grade should be reported.
 A single dash (-) indicates a severity grade is not available. Not all grades are appropriate for all AEs.
8

## Slide 9
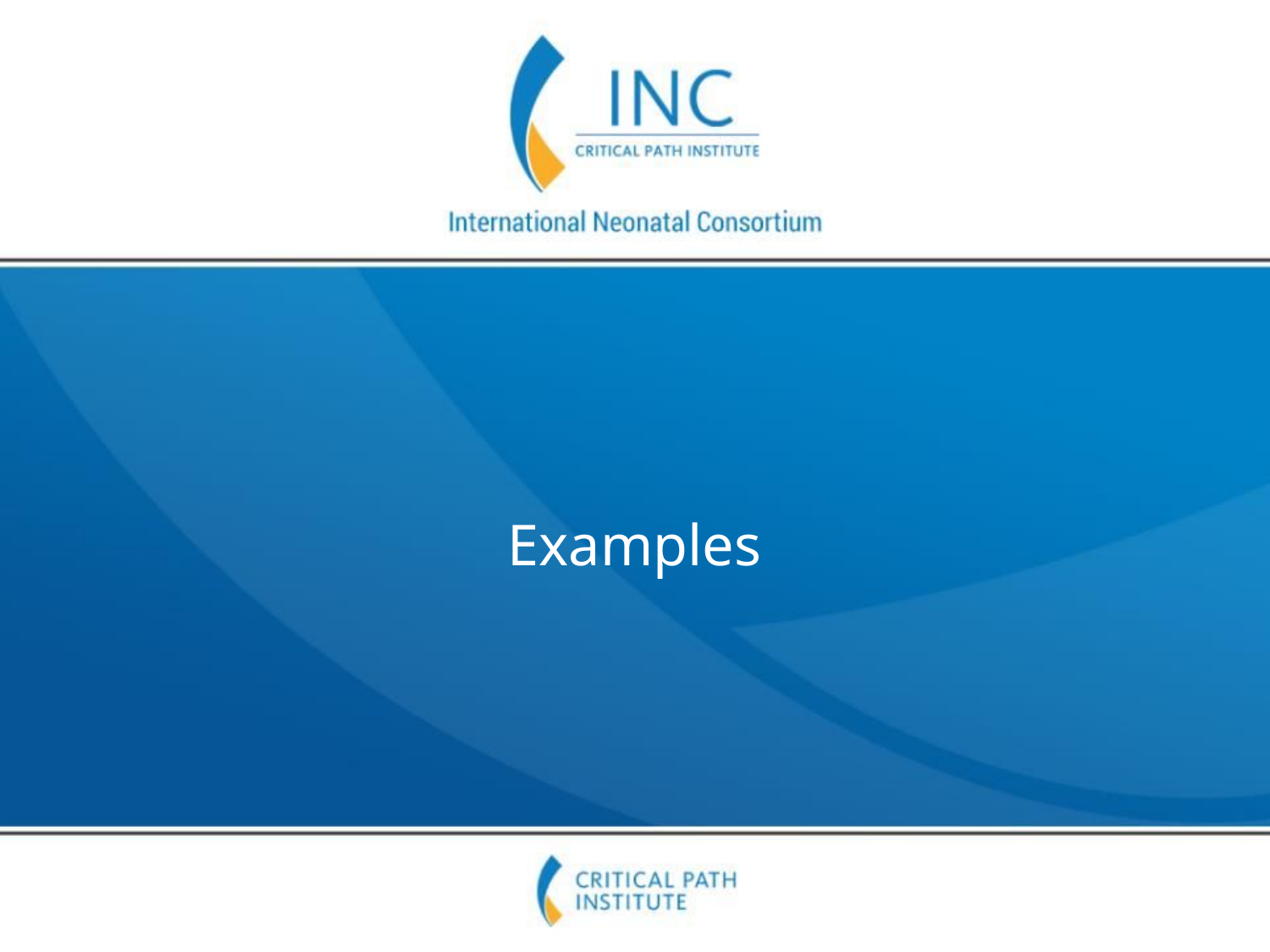

# Examples

## Slide 10
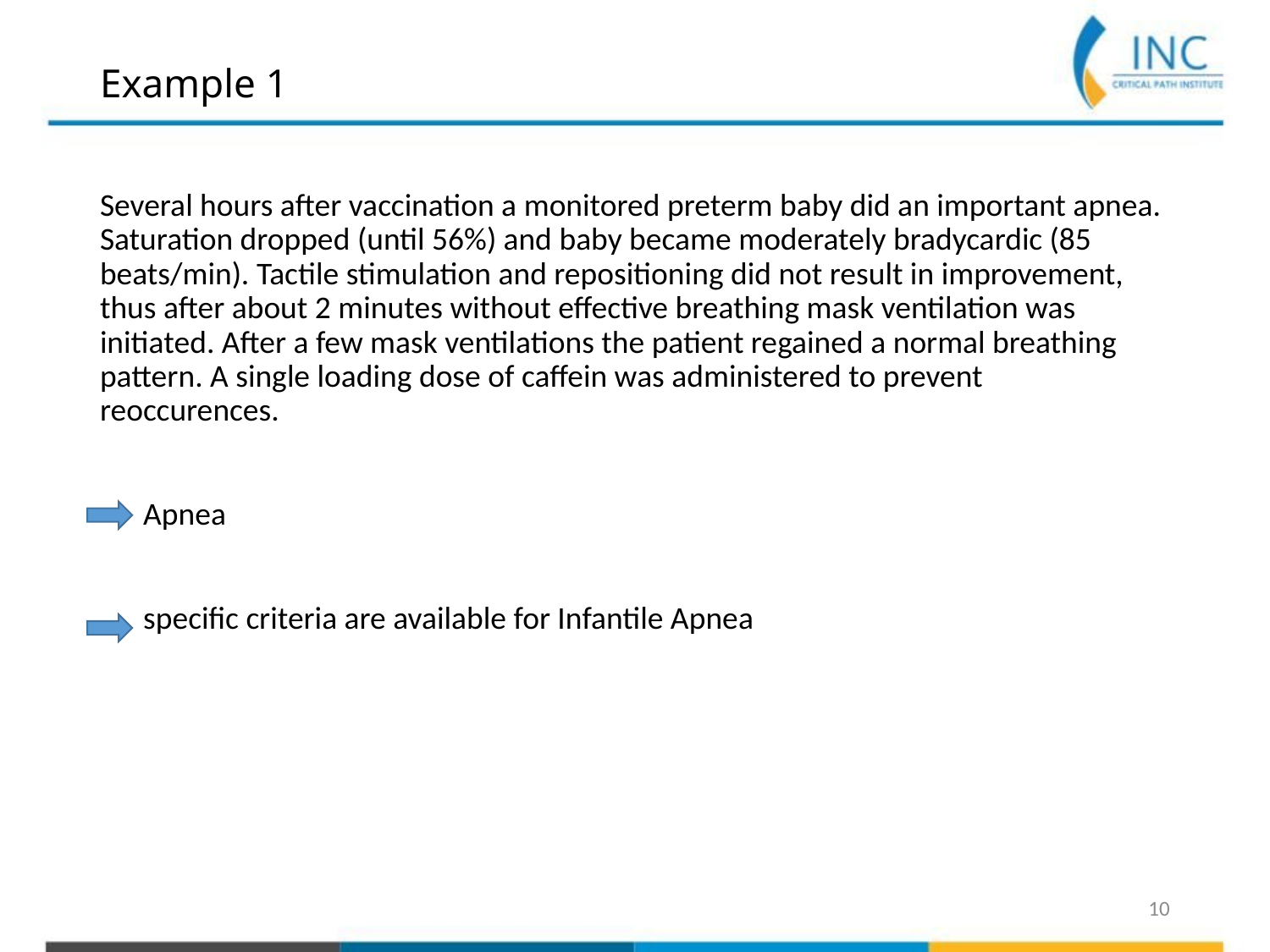

# Example 1
Several hours after vaccination a monitored preterm baby did an important apnea. Saturation dropped (until 56%) and baby became moderately bradycardic (85 beats/min). Tactile stimulation and repositioning did not result in improvement, thus after about 2 minutes without effective breathing mask ventilation was initiated. After a few mask ventilations the patient regained a normal breathing pattern. A single loading dose of caffein was administered to prevent reoccurences.
 Apnea
 specific criteria are available for Infantile Apnea
10

## Slide 11
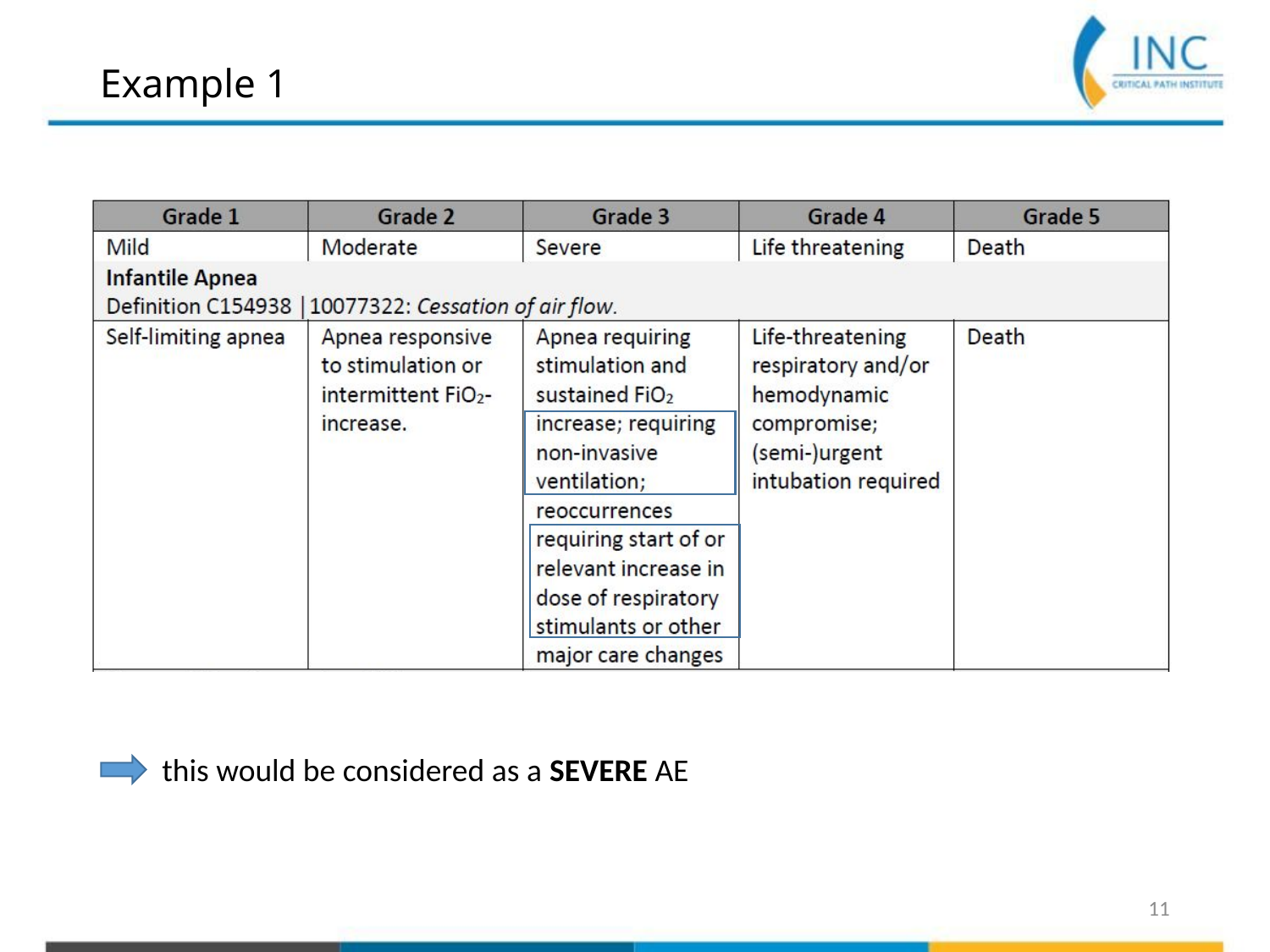

# Example 1
this would be considered as a SEVERE AE
11

## Slide 12
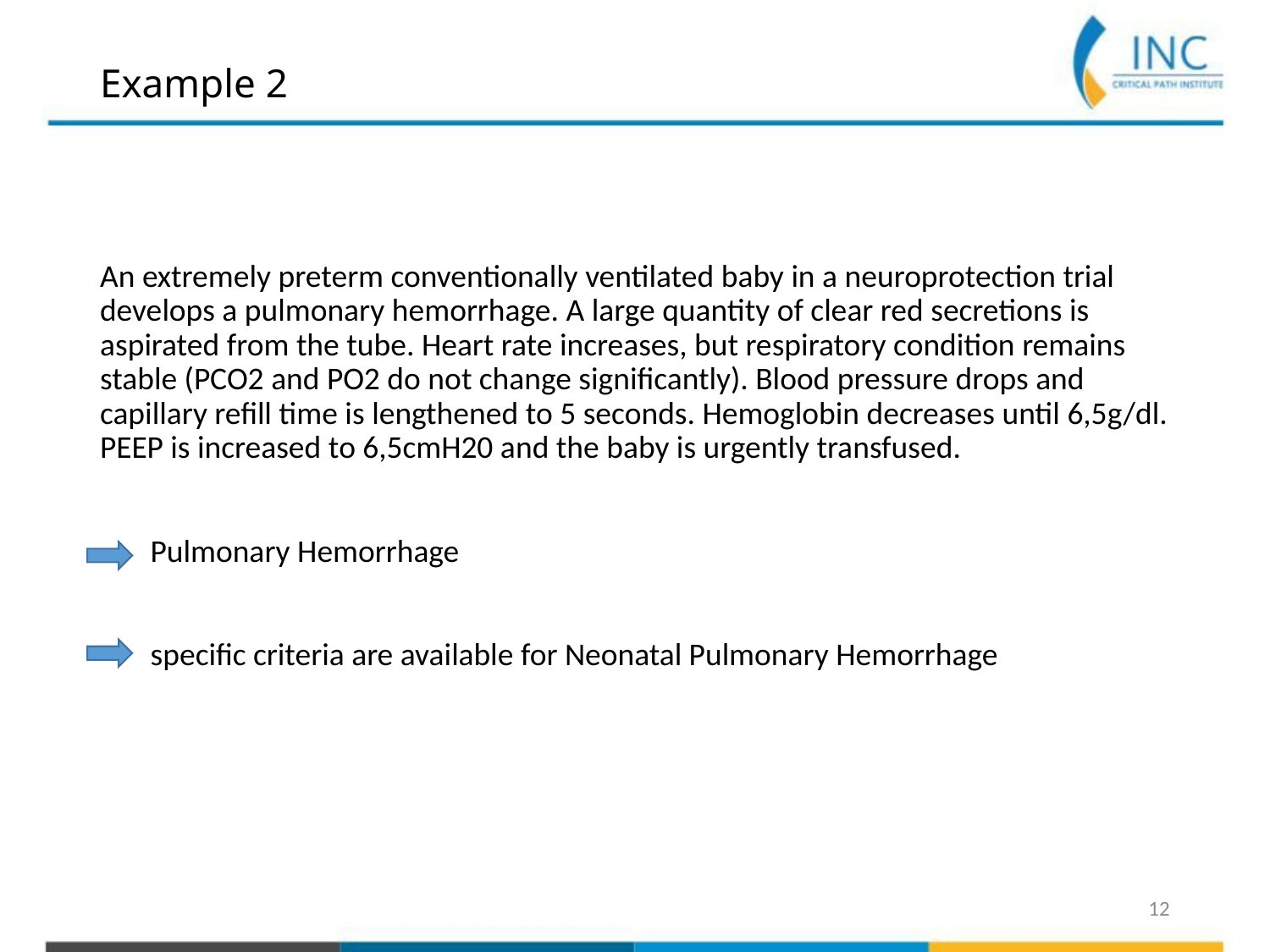

# Example 2
An extremely preterm conventionally ventilated baby in a neuroprotection trial develops a pulmonary hemorrhage. A large quantity of clear red secretions is aspirated from the tube. Heart rate increases, but respiratory condition remains stable (PCO2 and PO2 do not change significantly). Blood pressure drops and capillary refill time is lengthened to 5 seconds. Hemoglobin decreases until 6,5g/dl. PEEP is increased to 6,5cmH20 and the baby is urgently transfused.
 Pulmonary Hemorrhage
 specific criteria are available for Neonatal Pulmonary Hemorrhage
12

## Slide 13
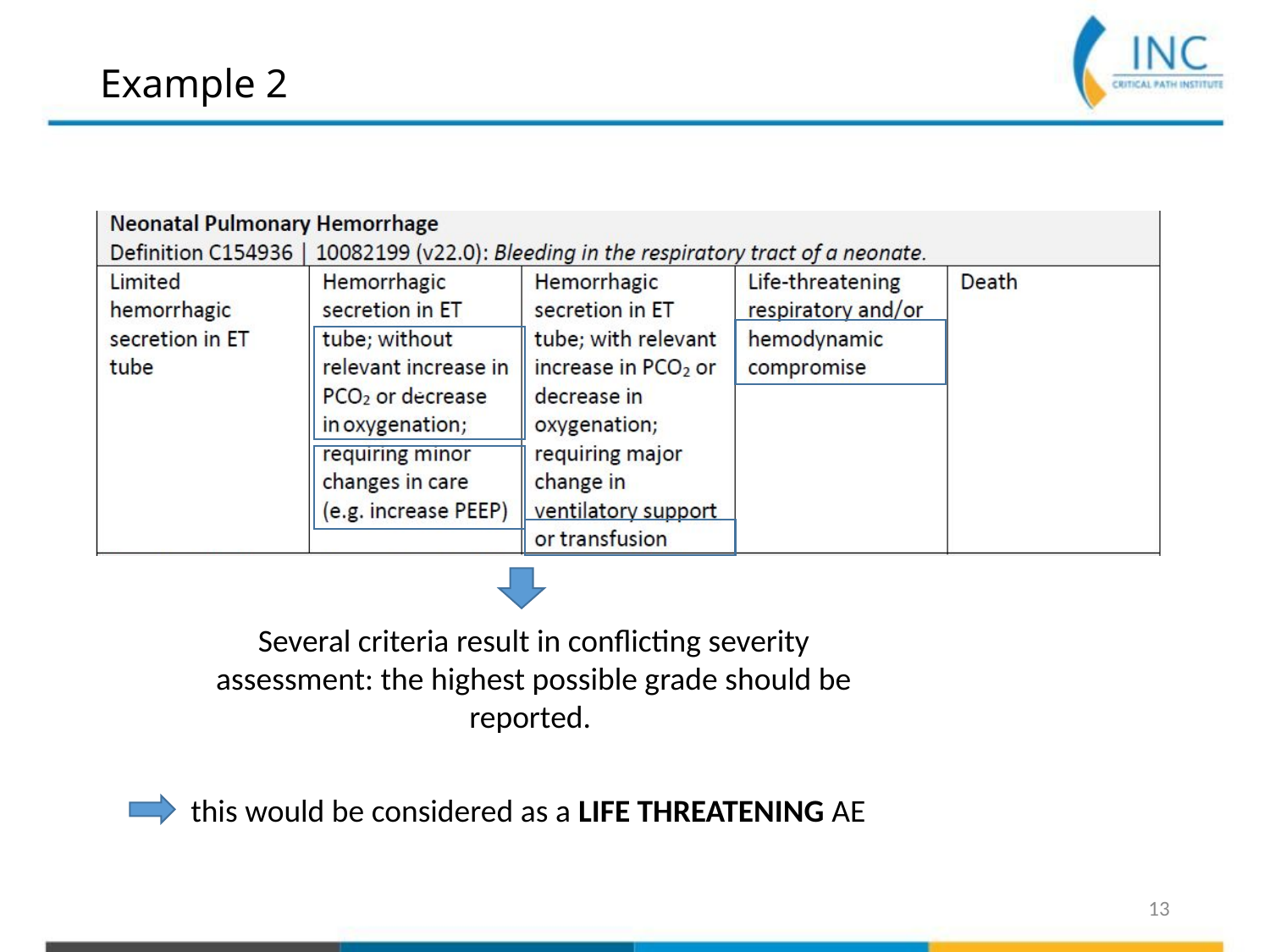

# Example 2
c
Several criteria result in conflicting severity assessment: the highest possible grade should be reported.
this would be considered as a LIFE THREATENING AE
13

## Slide 14
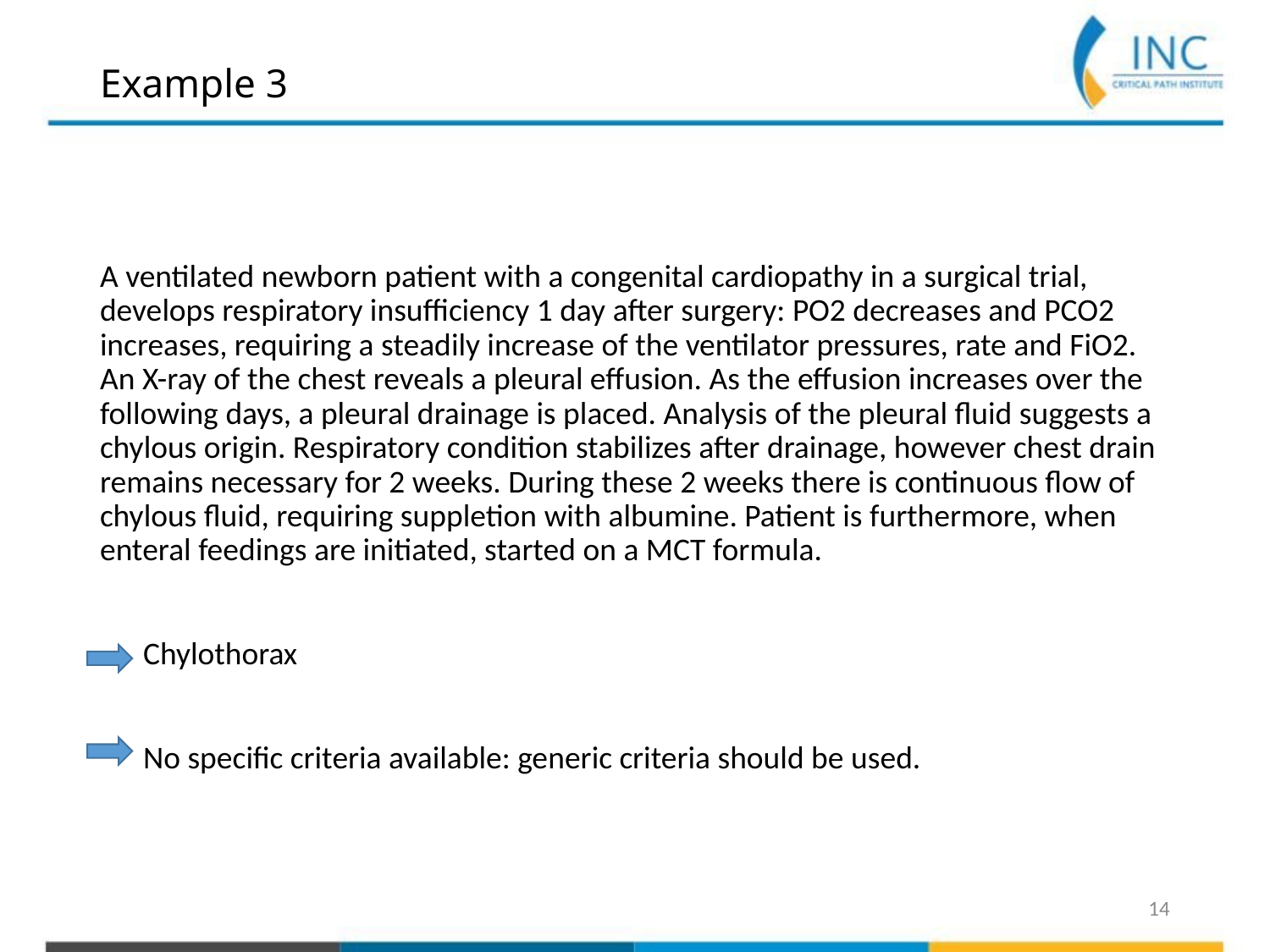

# Example 3
A ventilated newborn patient with a congenital cardiopathy in a surgical trial, develops respiratory insufficiency 1 day after surgery: PO2 decreases and PCO2 increases, requiring a steadily increase of the ventilator pressures, rate and FiO2. An X-ray of the chest reveals a pleural effusion. As the effusion increases over the following days, a pleural drainage is placed. Analysis of the pleural fluid suggests a chylous origin. Respiratory condition stabilizes after drainage, however chest drain remains necessary for 2 weeks. During these 2 weeks there is continuous flow of chylous fluid, requiring suppletion with albumine. Patient is furthermore, when enteral feedings are initiated, started on a MCT formula.
 Chylothorax
 No specific criteria available: generic criteria should be used.
14

## Slide 15
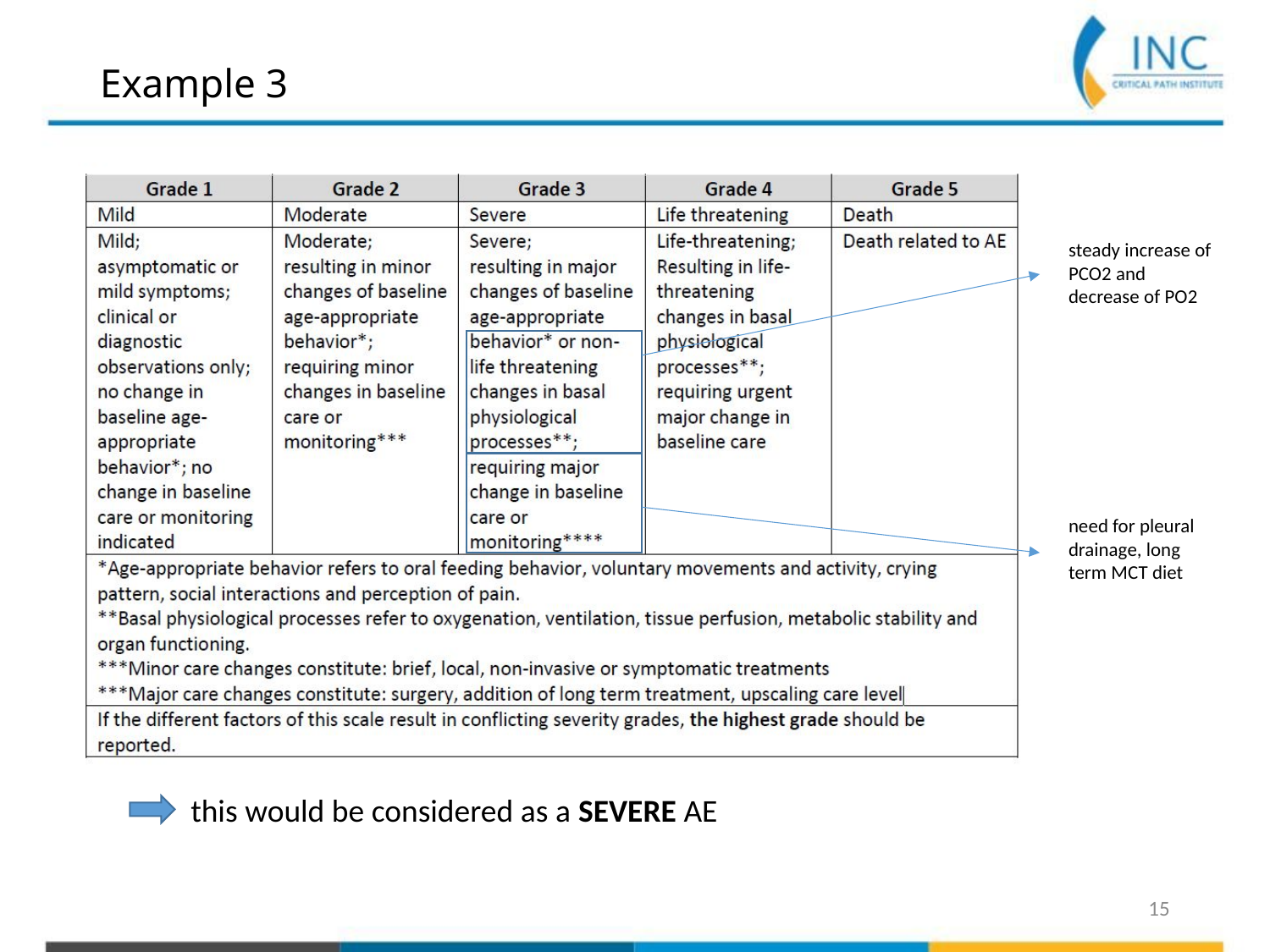

# Example 3
steady increase of PCO2 and decrease of PO2
need for pleural drainage, long term MCT diet
this would be considered as a SEVERE AE
15

## Slide 16
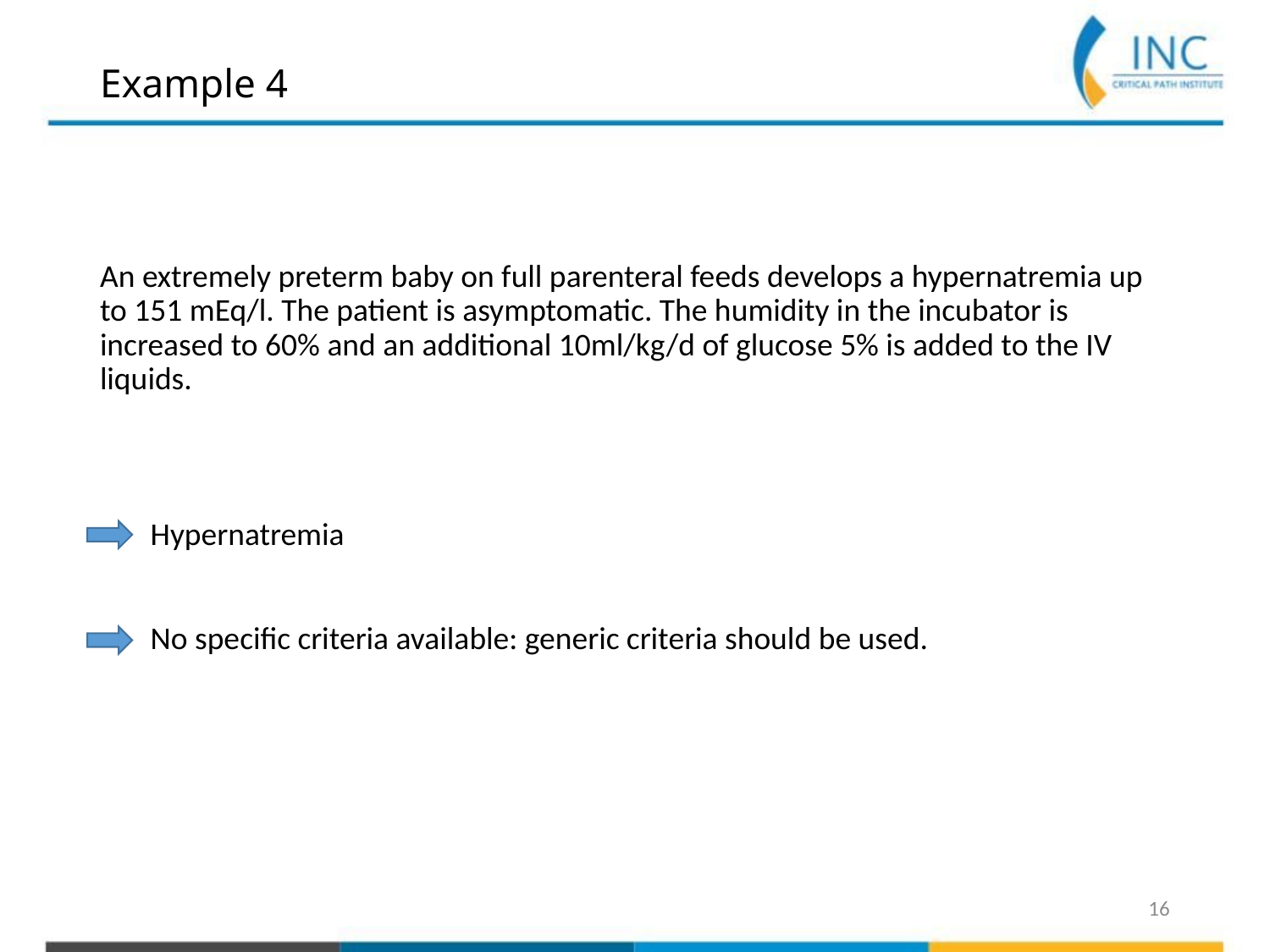

# Example 4
An extremely preterm baby on full parenteral feeds develops a hypernatremia up to 151 mEq/l. The patient is asymptomatic. The humidity in the incubator is increased to 60% and an additional 10ml/kg/d of glucose 5% is added to the IV liquids.
 Hypernatremia
 No specific criteria available: generic criteria should be used.
16

## Slide 17
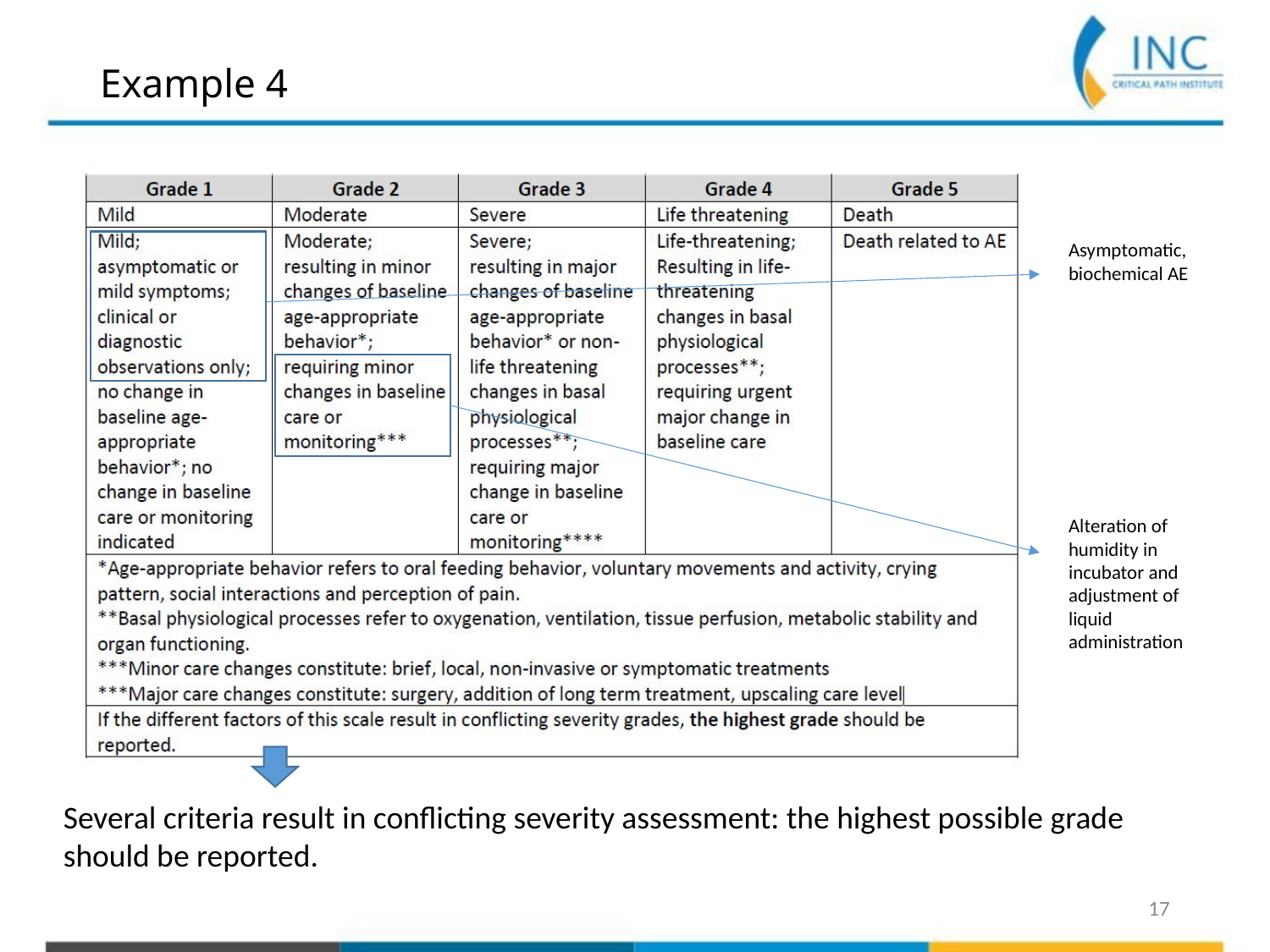

# Example 4
Asymptomatic, biochemical AE
Alteration of humidity in incubator and adjustment of liquid administration
Several criteria result in conflicting severity assessment: the highest possible grade should be reported.
17

## Slide 18
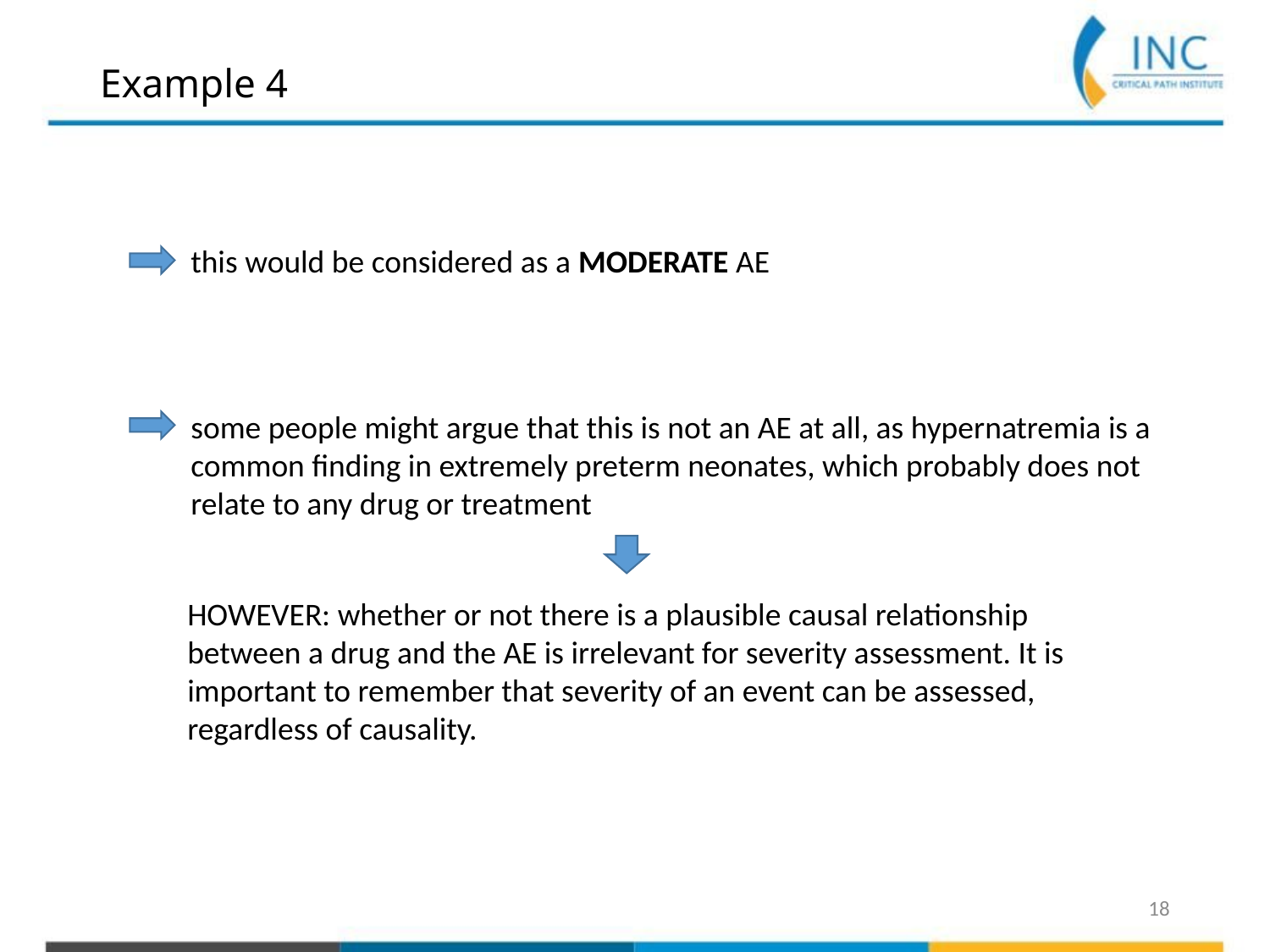

# Example 4
this would be considered as a MODERATE AE
some people might argue that this is not an AE at all, as hypernatremia is a common finding in extremely preterm neonates, which probably does not relate to any drug or treatment
HOWEVER: whether or not there is a plausible causal relationship between a drug and the AE is irrelevant for severity assessment. It is important to remember that severity of an event can be assessed, regardless of causality.
18

## Slide 19
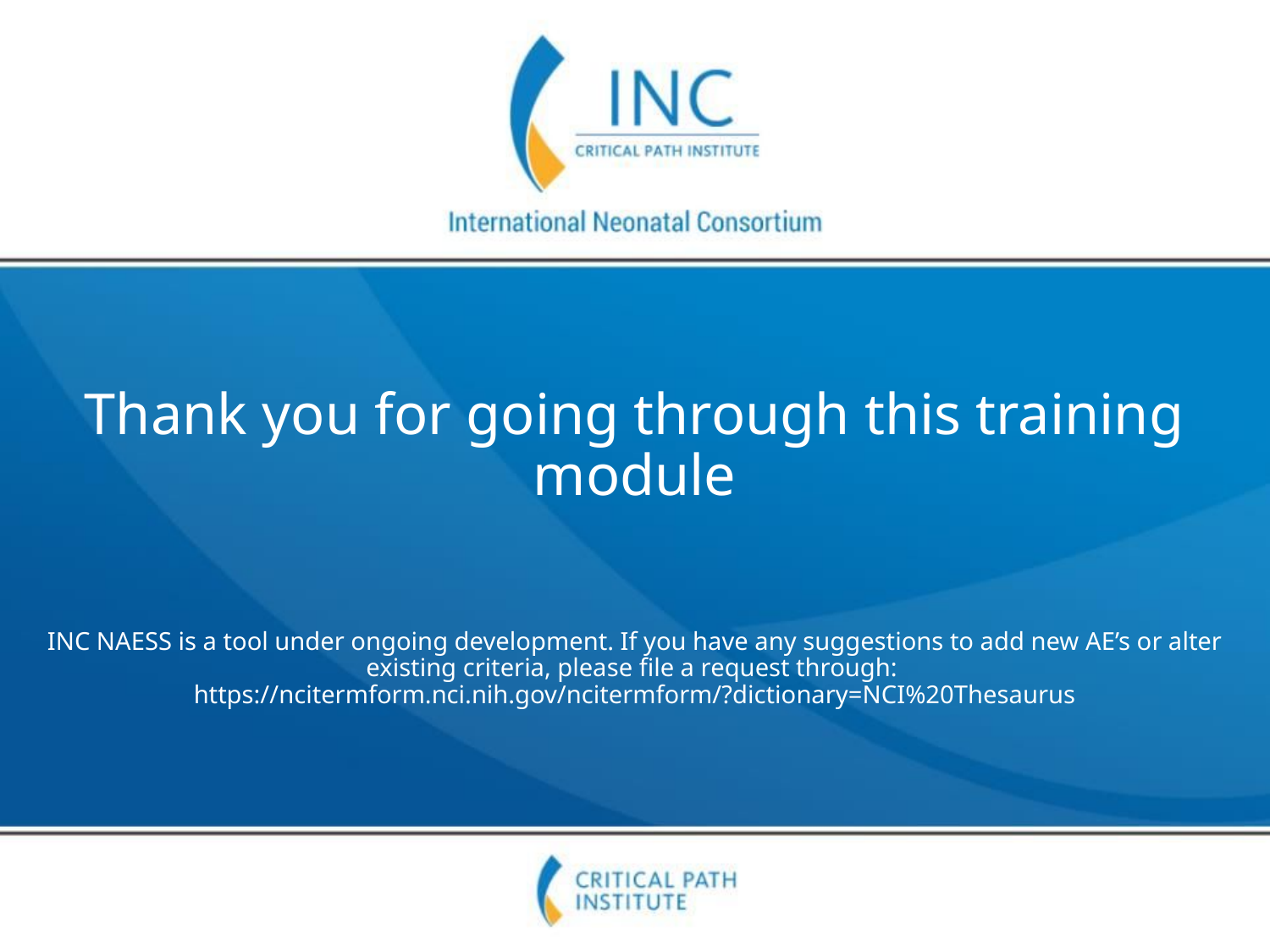

# Thank you for going through this training module INC NAESS is a tool under ongoing development. If you have any suggestions to add new AE’s or alter existing criteria, please file a request through: https://ncitermform.nci.nih.gov/ncitermform/?dictionary=NCI%20Thesaurus
